# Comparative physiology and transcriptome response patterns in cold-tolerant and cold-sensitive varieties of *Solanum melongena*

**DOI:** 10.1186/s12870-024-04922-y

**Published:** 2024-04-09

**Authors:** Peng cai, Yanhong lan, Fangyi Gong, Chun Li, Feng Xia, Yifan Li, Chao Fang

**Affiliations:** 1 Horticulture Research Institute, Sichuan Academy of Agricultural Sciences, Chengdu, 610066 China; 2 Vegetable Germplasm Innovation and Variety Improvement Key Laboratory of Sichuan Province, Chengdu, 610066 China; 3 Sichuan Province Engineering Technology Research Center of Vegetables, Chengdu, 611934 China

**Keywords:** *Solanum melongena*, Cold stress, Transcriptome, Chemometrics analysis, Response model co-expression network

## Abstract

**Background:**

Climate change has led to severe cold events, adversely impacting global crop production. Eggplant (*Solanum melongena L.*), a significant economic crop, is highly susceptible to cold damage, affecting both yield and quality. Unraveling the molecular mechanisms governing cold resistance, including the identification of key genes and comprehensive transcriptional regulatory pathways, is crucial for developing new varieties with enhanced tolerance.

**Results:**

In this study, we conducted a comparative analysis of leaf physiological indices and transcriptome sequencing results. The orthogonal partial least squares discriminant analysis (OPLS-DA) highlighted peroxidase (POD) activity and soluble protein as crucial physiological indicators for both varieties. RNA-seq data analysis revealed that a total of 7024 and 6209 differentially expressed genes (DEGs) were identified from variety “A” and variety “B”, respectively. Gene Ontology (GO) and Kyoto Encyclopedia of Genes and Genomes (KEGG) pathway enrichment of DEGs demonstrated that the significant roles of starch and sucrose metabolism, glutathione metabolism, terpenoid synthesis, and energy metabolism (sucrose and starch metabolism) were the key pathways in eggplant. Weighted gene co-expression network analysis (WGCNA) shown that the enrichment of numerous cold-responsive genes, pathways, and soluble proteins in the MEgrep60 modules. Core hub genes identified in the co-expression network included *POD*, membrane transporter-related gene *MDR1*, abscisic acid-related genes, growth factor enrichment gene *DELLA*, core components of the biological clock *PRR7*, and five transcription factors. Among these, the core transcription factor MYB demonstrated co-expression with signal transduction, plant hormone, biosynthesis, and metabolism-related genes, suggesting a pivotal role in the cold response network.

**Conclusion:**

This study integrates physiological indicators and transcriptomics to unveil the molecular mechanisms responsible for the differences in cold tolerance between the eggplant cold-tolerant variety “A” and the cold-sensitive variety “B”. These mechanisms include modulation of reactive oxygen species (ROS), elevation in osmotic carbohydrate and free proline content, and the expression of terpenoid synthesis genes. This comprehensive understanding contributes valuable insights into the molecular underpinnings of cold stress tolerance, ultimately aiding in the improvement of crop cold tolerance.

**Supplementary Information:**

The online version contains supplementary material available at 10.1186/s12870-024-04922-y.

## Background

Eggplant (*Solanum melongena L.*) holds a prominent position among vegetable crops cultivated in both the southern and northern regions of China. It has evolved into a vital industry ensuring a consistent supply of vegetables throughout the year, thereby contributing significantly to the augmentation of farmers’ income and supporting rural revitalization. The predominant method of eggplant cultivation in China relies on early spring planting. However, the vulnerability of early spring cultivation to low temperatures results in sluggish plant growth, flower and fruit drop, fruit deformities, and delayed fruit expansion speed [1]. These issues detrimentally impact both yield and economic returns. Enhancing the cold resistance of eggplants has thus emerged as a key objective in the breeding of early maturing eggplants.

Low-temperature stress manifests in two distinct forms: chilling injury (> 0 ℃) and freezing injury (≤ 0 ℃). These challenges are recognized as among the most destructive threats, exerting adverse effects on the plant life cycle, geographical distribution, and crop yield [2, 3]. The response of plants to low-temperature stress constitutes a complex regulatory system. When exposed to cold stress, plants initiate a series of physiological responses, including modifications to cell membrane lipid composition, clearance of ROS [4], and the maintenance of a steady-state balance in the cell membrane system [5]. Some studies have revealed that the exogenous addition of various chemicals, such as H_2_O_2_, abscisic acid (ABA), and methyl jasmonate (MeJA), can safeguard plants from cold damage. For instance, H_2_O_2_ stimulates the accumulation of plant hormones (ABA, MeJA, etc.), consequently enhancing the cold stress tolerance of tomatoes [6, 7]. The application of exogenous ABA increases the activity of wheat antioxidant enzymes, including catalase (CAT), superoxide dismutase (SOD), and POD, thereby mitigating cold damage [8]. Similarly, MeJA reinforces tomato cold resistance by elevating the activity of antioxidant enzymes, including CAT and POD, along with the expression of related genes [9]. Plant adaptation to cold stress also encompasses molecular-level changes, such as alterations in transcription, translation, and metabolic processes involving specific proteins, metabolites, and plant hormone levels.

The alterations in gene expression levels associated with the response to cold stress represent pivotal adaptive molecular mechanisms in plants combating adverse cold conditions. Notably, cold response genes, including C receptor binding factors (CBFs), inducers of CBF expression genes (*ICEs*) [10], cold-regulated (*COR*) genes [11], and other key regulators, are swiftly upregulated, contributing to heightened cold resistance [12]. Transcription factors linked to cold response, such as bHLHs, WRKYs, and MYBs, have been validated as crucial regulatory elements for cold response genes in model plant species like *Arabidopsis*, enhancing plant adaptability to cold [13–16]. Moreover, plants’ adaptation to cold stress encompasses multiple signal transduction pathways governing cold response gene expression, such as the abscisic acid signaling pathway [17], CBF expression (ICE)-C-repeat binding factor (CBF)-cold-responsive (COR) pathway [18], and mitogen-activated protein kinase signaling pathway [19]. These pathways influence the expression levels of genes regulating the rearrangement of plant secondary metabolites, particularly antioxidant metabolites like flavonoids and terpenoids. The cold-induced accumulation of polyphenolic compounds, for instance, enhances free radical scavenging activity and antioxidant capacity in *Brassica rapa* L. ssp. pekinensis, contributing to its cold tolerance [20]. However, such changes involve the expression of numerous related genes and the regulation of regulatory factors, making it challenging to unravel the intricate network of plant adaptation to cold solely by studying individual genes and metabolic pathways. The advent of high-throughput sequencing has facilitated the comprehensive exploration of the entire genome’s expression at the transcriptional level. Transcriptome sequencing has been conducted on various plants under low-temperature stress, encompassing model plants [21–24], horticultural plants [25], vegetable crops [20], and trees [26]. These studies offer insights into how transcriptional changes respond to cold stress, leading to the identification of numerous cold response genes across different crops. However, it is essential to recognize that distinct species may exhibit varying cold response mechanisms, necessitating ongoing exploration of regulatory pathways and genes involved in cold response across diverse plant species.

Eggplant, native to tropical Asia, has now gained global popularity as a commercially significant economic crop. However, due to incomplete genomic information and a lack of expression profile data, research on low-temperature tolerance in eggplants has traditionally focused on enhancing cultivation management measures and basic physiological and biochemical aspects. Unfortunately, this approach did not fundamentally address the urgent need for low-temperature-tolerant varieties in production. Advances in technology, coupled with the widespread use of modern molecular biology methods, high-throughput sequencing, and genetic engineering techniques, have allowed for the preliminary identification of several genes associated with regulating low-temperature responses in eggplants [27–29]. While existing research has made progress, the depth of exploration varies, and there is a scarcity of reports concerning gene function analysis and regulatory mechanisms. A prior study by Yang et al. analyzed the transcriptome of eggplants under cold stress, identifying some DEGs related to cold stress, such as cold-inducible proteins, genes associated with hormone signal transduction, and osmoregulation proteins [29]. However, the metabolic pathways associated with these DEGs were not thoroughly examined. In this study, we conducted a comprehensive investigation into the phenotypic and physiological differences between the cold-tolerant “E7135” and cold-sensitive “E7142” eggplant varieties. Employing phenotype identification and transcriptomics methods, we delved into potential mechanisms underlying eggplant cold responses at physiological, biochemical, transcriptional, and metabolic levels, constructing a cold stress transcriptional regulatory network. The findings of this research serve as a foundation for a deeper understanding of the molecular mechanisms governing eggplant adaptation to cold stress and identifying genes with the potential to enhance the low-temperature tolerance of early maturing eggplants.

## Results

### Physiological response of eggplant under cold stress treatment

**Fig. 1:**
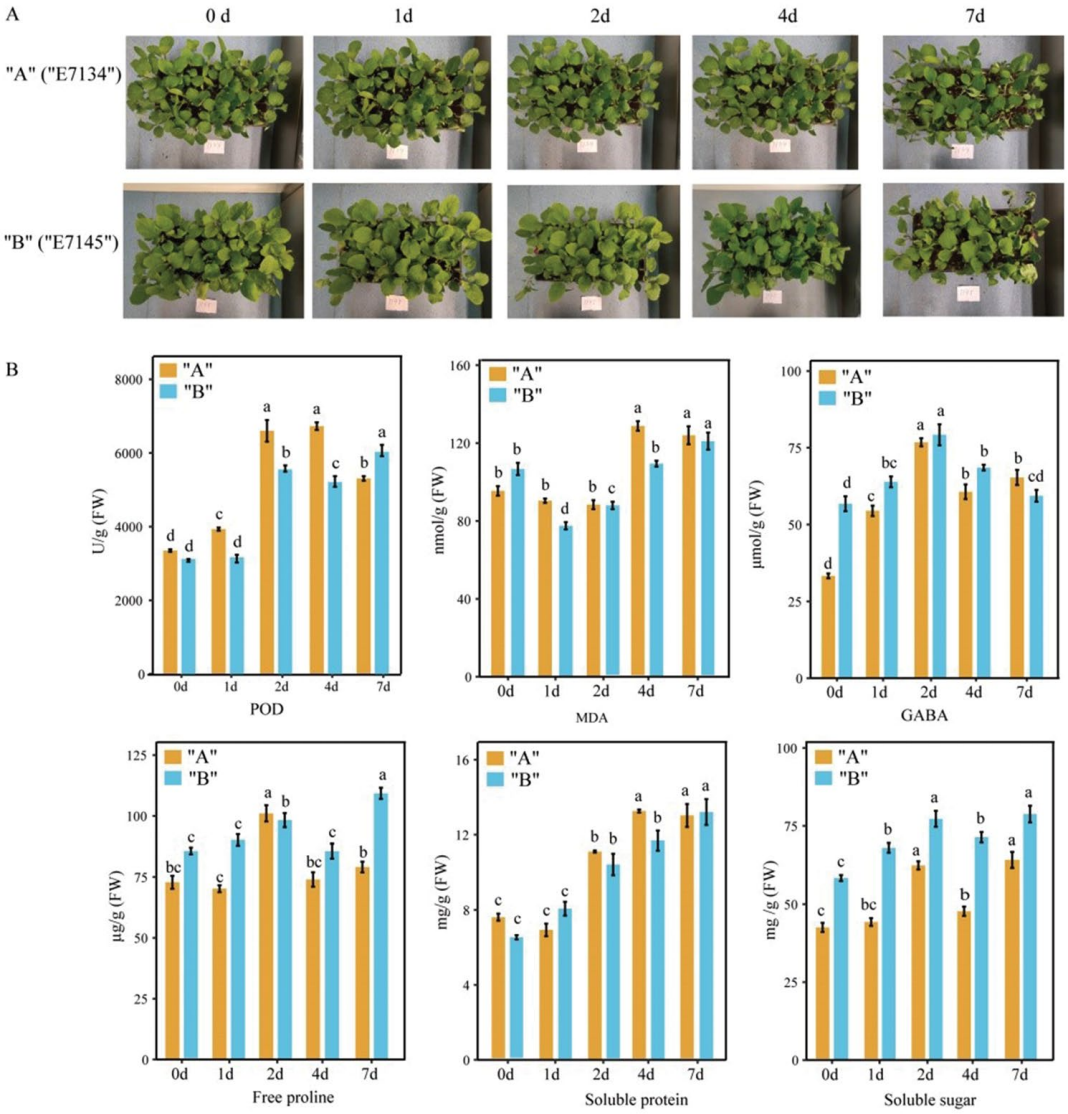
Physiological response of eggplant to cold stress. (**A**) Phenotypic changes in eggplant seedlings under cold stress treatment (**B**) Physiological data of eggplant under cold stress treatment

This study aimed to compare the cold resistance of the cold-tolerant “E7134” (“A”) eggplant variety and the cold-sensitive “7145” (“B”) variety. Morphological changes of two eggplant varieties “A” and “B” under 5 ℃ cold stress exhibited significant differences (Fig. 1a). Specifically, leaves of “B” began dehydrating at 4 d, intensifying by 7 d, while “A” showed dehydrating only after 7 d. “B” displayed leaf curling after 4 d, contrasting with “A,” which showed no notable changes, confirming “A” as cold-resistant and “B” as cold-sensitive.

To investigate To comprehend cellular responses to oxidative and osmotic damage under cold stress, we measured the activity of POD and the content of osmoregulation-related components, including malondialdehyde (MDA), γ-aminobutyric acid (GABA), free proline, soluble protein, and soluble sugar in eggplant leaves (Fig. 1b). The POD activity in sample “A” exhibited a significant increase after 1 d of cold stress at 5 ℃, escalating by 1.17 times (1 d) and 2.01 times (2 d) compared to the control group (0 d), respectively. Conversely, the POD activity in sample “B” showed no significant changes on the first day but increased by 1.77-fold after 2 d under cold stress, with a notable decrease at 4 d. More importantly, the POD activity of sample “A” surpassed that of sample “B” under cold stress. The MDA content in sample “A” remained largely unchanged at 2 d post-cold stress, peaking at 4 d. In contrast, the MDA content in sample “B” consistently decreased over time, rising significantly after the 4th d but consistently remaining lower than that of sample “A”. After 2 d of cold stress, the GABA content in both samples “A” and “B” peaked, with sample “B” exhibiting slightly higher levels during the stress period. The free proline content in samples “A” and “B” gradually increased 2 d before cold stress, rising by nearly 1.35 and 1.16 times compared to the control group (0 d), respectively. On the 4th d, a significant decrease was observed, followed by an increase. The soluble protein content in sample “A” reached its peak at the 4th d under cold stress, nearly 1.21 times higher than sample “B”. After 1 d of cold stress, the soluble sugar content in sample “B” significantly increased. In contrast, the soluble sugar content in sample “A” was significantly lower than that of sample “B” during the entire cold stress period. These findings underscore significant differences in cold resistance between the two eggplant varieties, indicating distinct molecular regulatory mechanisms.

### Chemometrics analysis based on physiological index data

**Fig. 2:**
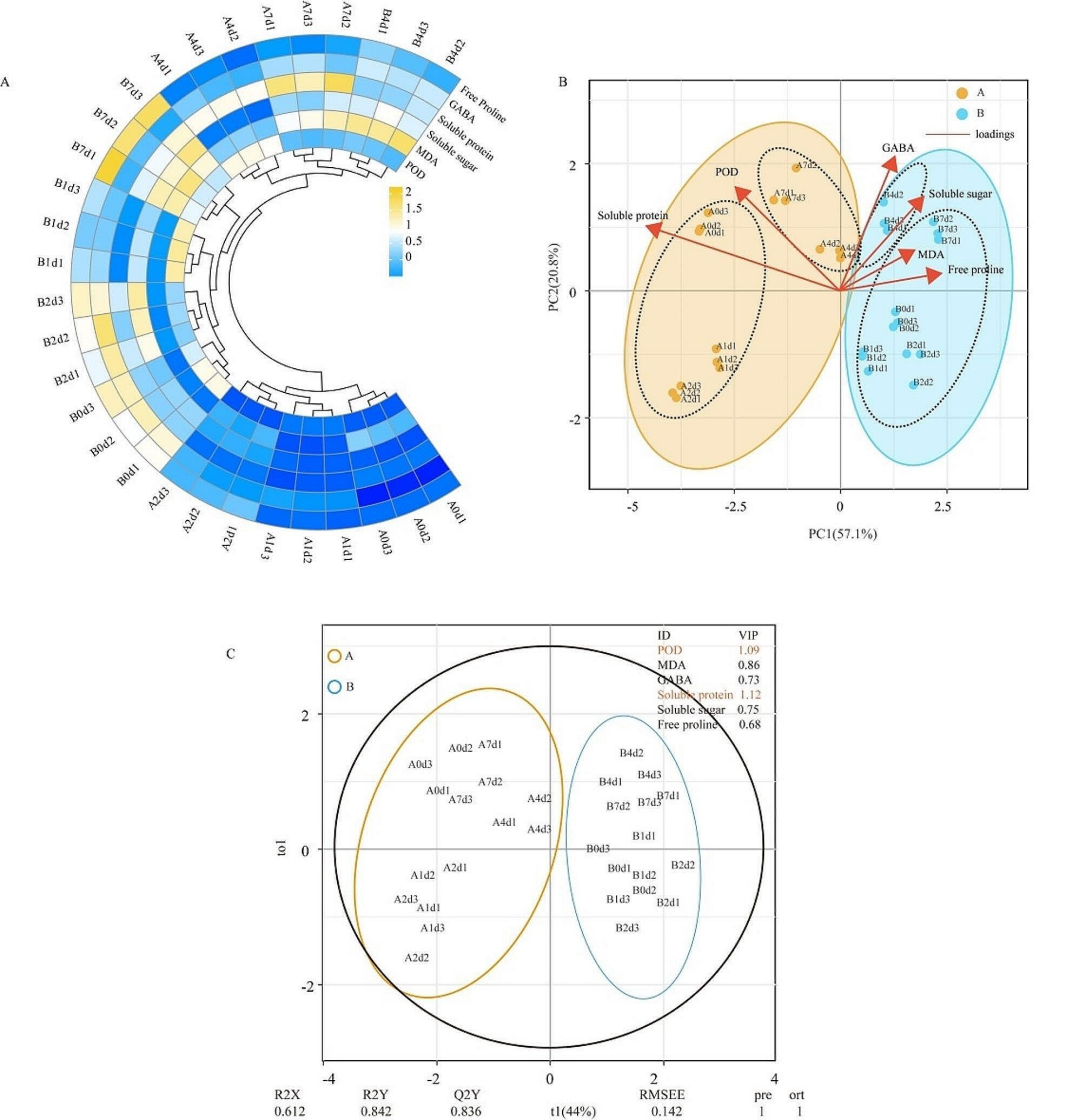
Comprehensive analysis of physiological indicators of eggplant under cold stress treatment (**A**) Cluster heat map based on physiological indicators. (**B**) Principal component load map based on physiological indicators. (**C**) Orthogonal partial least squares discriminant analysis based on physiological indicators

First, physiological data were standardized for Chemometrics analysis, and the results were clustered with cold stress samples (Fig. 2a). The 30 samples were categorized into three groups: “A” 0d, “A” 1d, “A” 2d (treated with 2 d of cold stress) forming the first category, “B” 0d, “B” 1d, “B” 2d, and “B” 7d forming the second category, and “A” 4d, “A” 7d, and “B” 4d forming the third category. Next, principal component analysis (PCA) was employed to discern grouping characteristics and physiological indicators of the samples. PC1, which accounted for 57.1% of the total variance, distinctly separated the samples: “A” 0d - “A” 7d (represented by orange) on the left and “B” 0d − 7d (represented by blue) on the right (Fig. 2b). Sample “A” was further divided into two groups (“A” 0d − 2d, “A” 4d − 7d) in PC2 (20.8%), while sample “B” was divided into two groups (“B” 0d − 7d, “B” 4d). Thus, variety differences were distinguishable by PC1, and differences induced by cold stress treatment were discernible by PC2. Moreover, the positive load of PC1 encompassed GABA, MDA, free proline, and soluble sugar, while the negative load comprised soluble protein and POD. All physiological indicators of PC2 were positive loads. Finally, OPLS-DA was employed to evaluate the crucial physiological indicators for the two varieties under low-temperature stress. The Variable Importance in Projection (VIP) threshold served as a measure of the impact intensity of physiological indicators. Researchers identified that POD (VIP = 1.09) and soluble protein (VIP = 1.12) emerged as important physiological indicators for both varieties under low-temperature stress (Fig. 2c).

Gene expression profiling of eggplant under cold stress treatment.

**Fig. 3:**
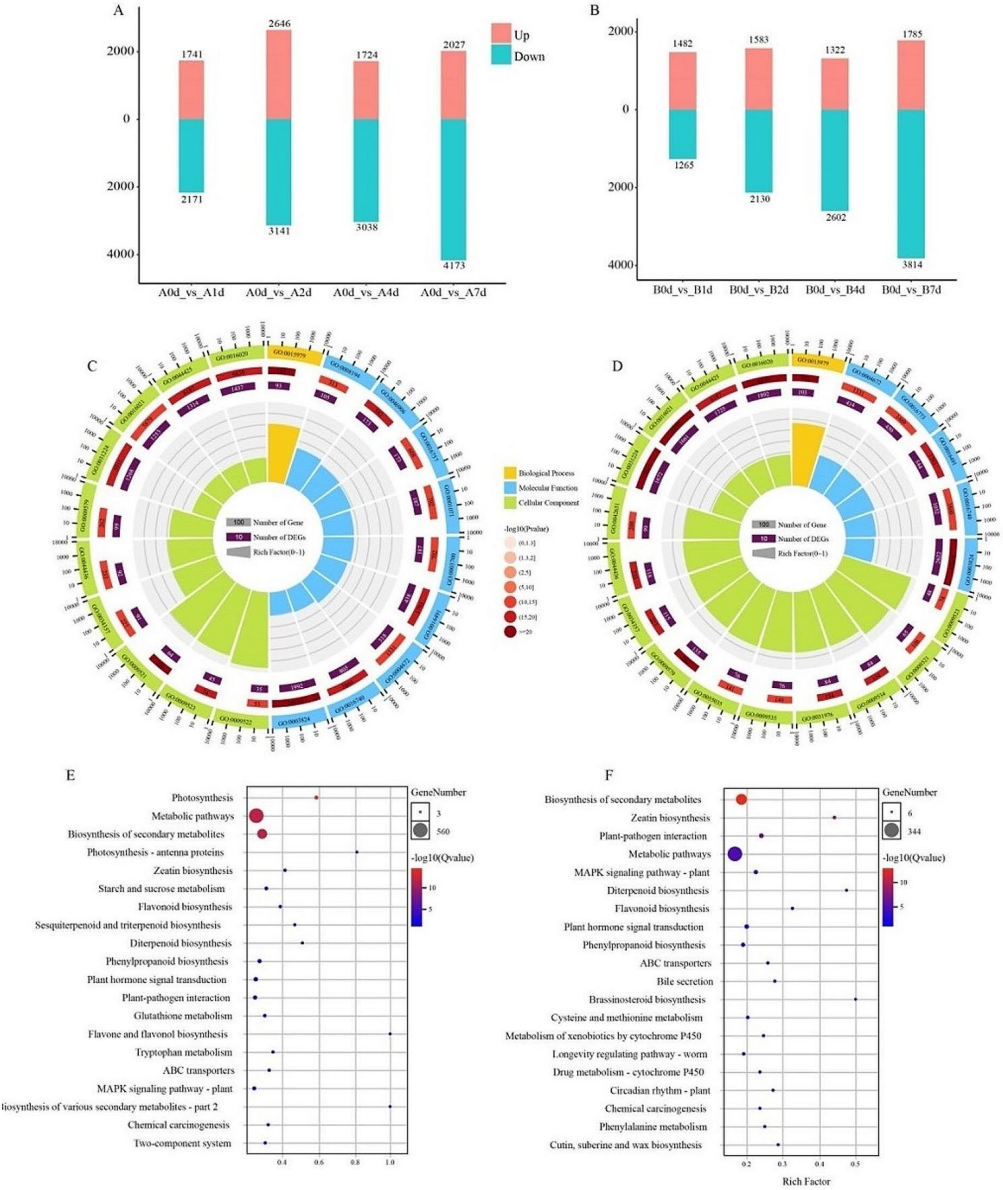
Transcriptome analysis of A and B under cold stress. (**A**)(**B**) The number of DEGs in different comparison groups. (**C**)(**D**) GO analysis of DEGs in A and B, respectively. (**E**)(**F**) KEGG analysis of DEGs in A and B, respectively

To analyze transcriptional changes between two eggplant cultivars under cold stress, designated as “A” and “B” both under cold stress treatment at five time points: before (CK, 0 d) and after exposure to 5 °C (1d, 2d, 4d, and 7d), with three biological replicates per sample. A total of 434.65 Gb of clean data were obtained from 30 samples, each reaching 6.17 Gb. All Q30 values surpassed 93.02%, attesting to the reliability of the sequencing results (Table S1). Table S2 showed detailed annotation information for unigenes containing Nonredundant (NR), Gene Ontology (GO), Kyoto Encyclopedia of Genes and Genomes (KEGG), and Non supervised Orthologous Groups (NOG) databases (Table S2). The sample heatmap analysis indicated correlation coefficients exceeding 0.8 for the treatment groups in each variety (Fig. S1). Differentially expressed genes (DEGs) were identified based on transcript abundance, using error detection rate < 0.01 and fold change ≥ 2 as thresholds. Both “A” and “B” exhibited similar and significant changes in their response to cold stress at different time points, with 7024 and 6209 DEGs identified, respectively (Fig. S2). Prior to cold treatment (CK, 0 d), “A” and “B” displayed 1741 and 1482 highly expressed genes, respectively, indicating genotype-related gene expression changes (Fig. 3a, b). “A” exhibited a larger number of DEGs from 1 d to 2 d, while “B” showed fewer upregulated and downregulated genes during this period. Specifically, “A” had 2646, 1724, and 2027 upregulated genes and 3143, 3038, and 4173 downregulated genes at each respective time point. In contrast, “B” showed 1583, 1322, and 1785 upregulated genes and 2130, 2602, and 3814 downregulated genes, respectively (Fig. 3a). Comparatively, “A” demonstrated a higher number of activated genes than “B,” indicating distinct responses between tolerant and sensitive varieties, with “A” exhibiting greater adaptability to cold stress. The number of shared genes significantly increased at 7 d in both “A” and “B”. Intriguingly, the shared genes in “A” and “B” exhibited different expression patterns during the 1–4 d stage of the cold stress response activation.

**Fig. 4:**
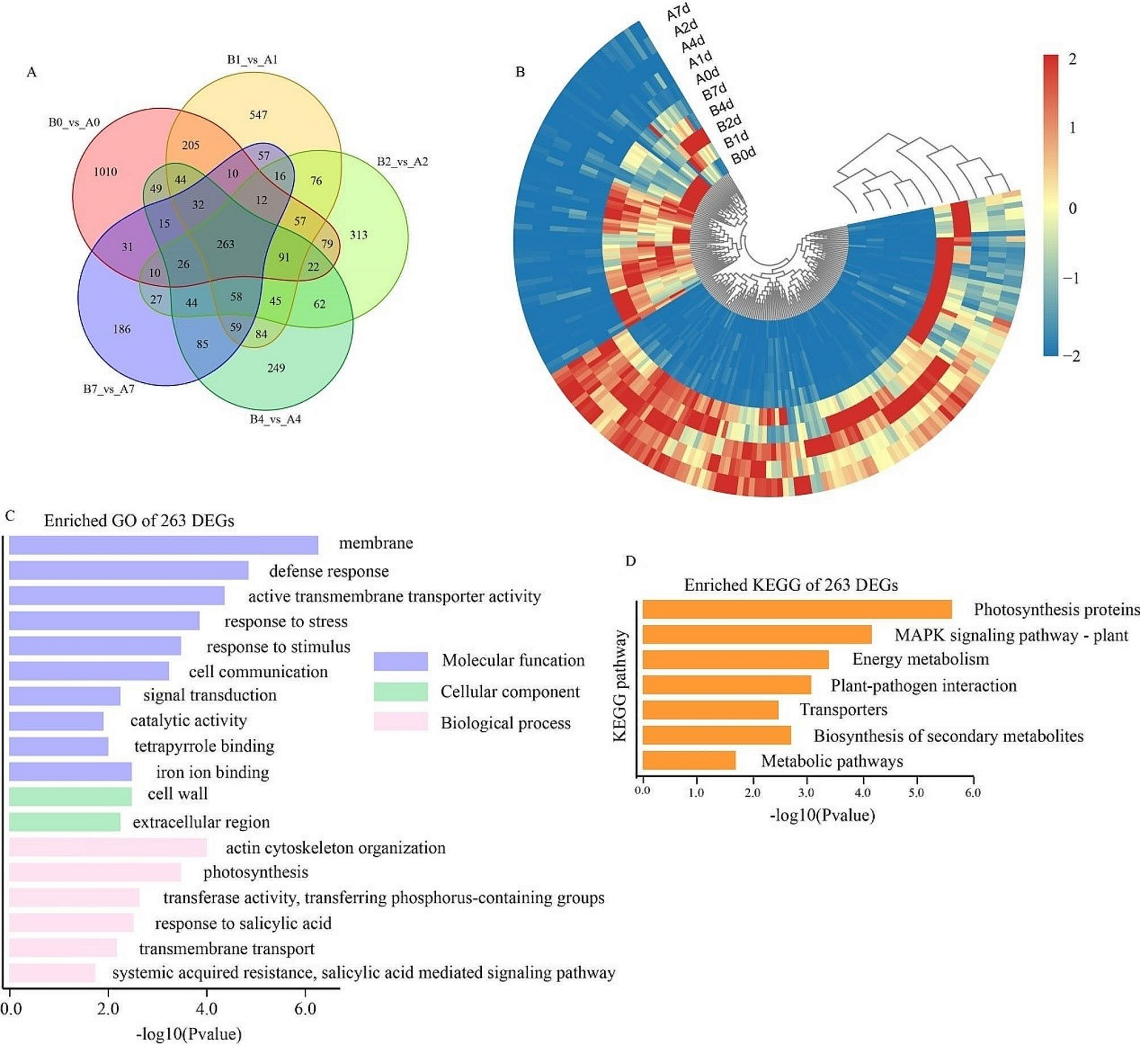
Analysis results of shared genes at different processing time points in different varieties. (**A**) Wayne Result Analysis of DEG in cold stressed seedlings; (**B**) The hierarchical expression spectrum of 263 DEGs shared in A and B. C0 and (D), GO (**C**) and KEGG enrichment (**D**) of 263 DEGs

### Transcriptome differences between genes with known cold response mechanisms and core metabolic pathways

All samples underwent GO term analysis, revealing significant enrichment in catalytic activity, membrane components, transport activity, and responses to stress. Notably, the number of DEGs enriched in membrane components and transport activity was higher in variety “A” compared to “B”. Variety “A” specifically exhibited enrichment in processes related to the cell wall, non-protoplast, and secondary metabolism. On the other hand, variety “B” showed specific enrichment in oxidoreductase activity, protein serine/threonine kinase activity, and terpene synthase activity (Fig. 3c, d). These results suggest that “A” enhances cold tolerance by fortifying cell structure (membrane components, cell walls) and secondary metabolism, facilitating plant growth under adverse conditions. In contrast, “B” primarily adapts to cold stress by enhancing kinase activity, showcasing a comparatively more focused response than “A”.

To delve deeper into the function of eggplant DEGs in response to low-temperature stress, KEGG pathway enrichment analysis was employed to identify biological pathways involved in cold stress. A Q value of ≤ 0.05 was used as the truncation criterion, revealing that compared to variety “B”, variety “A” exhibited a greater number of enriched metabolic pathways (Fig. 3e, f). Both varieties showed significant enrichment in metabolic pathways, synthesis of secondary metabolites, plant hormone signal transduction, and the mitogen-activated protein kinase (MAPK) signaling pathway under cold stress. Notably, starch and sucrose metabolism, synthesis of diterpenoid and tetraterpene compounds, and the glutathione biosynthesis pathway were specifically enriched in variety “A”, while DEGs of variety “B” were specifically enriched in brassinosteroid biosynthesis and plant circadian rhythm. This implies that, compared to “B”, variety “A” demonstrates a more diverse pathway for responding to cold stress.

To investigate the effects of cold stress on conserved responses within and between varieties, a Venn analysis was conducted (Fig. 4a). The results indicated that 263 DEGs were shared among the varieties, with nearly half of them highly expressed in “A” (Fig. 4b). GO enrichment analysis of these 263 DEGs revealed a higher proportion of genes related to membrane, transmembrane transport protein activity, response to stress, iron ion binding, and catalytic activity in molecular functional categories. Additionally, biological processes such as photosynthesis and transmembrane transport was enriched (Fig. 4c). KEGG enrichment analysis showed involvement in photosynthesis, biosynthesis of secondary metabolites, MAPK signaling pathway, and energy metabolism (Fig. 4d). These results indicate that ion transport, photosynthesis, and energy metabolism contribute to the conservative cold stress response of eggplant seedlings. While there are similarities in the expression patterns of shared genes, the differences suggest that cold stress response pathways have become more diverse.

### Response of core metabolic pathways to cold stress

To delve into the nuanced effects of cold stress on the core metabolism of eggplant, the transcriptional abundance of key genes in two varieties, involved in starch and sucrose metabolism, synthesis of diterpenoids and tetraterpenes, glutathione synthesis metabolic, photosynthesis, and chlorophyll degradation pathways, were compared.

**Fig. 5:**
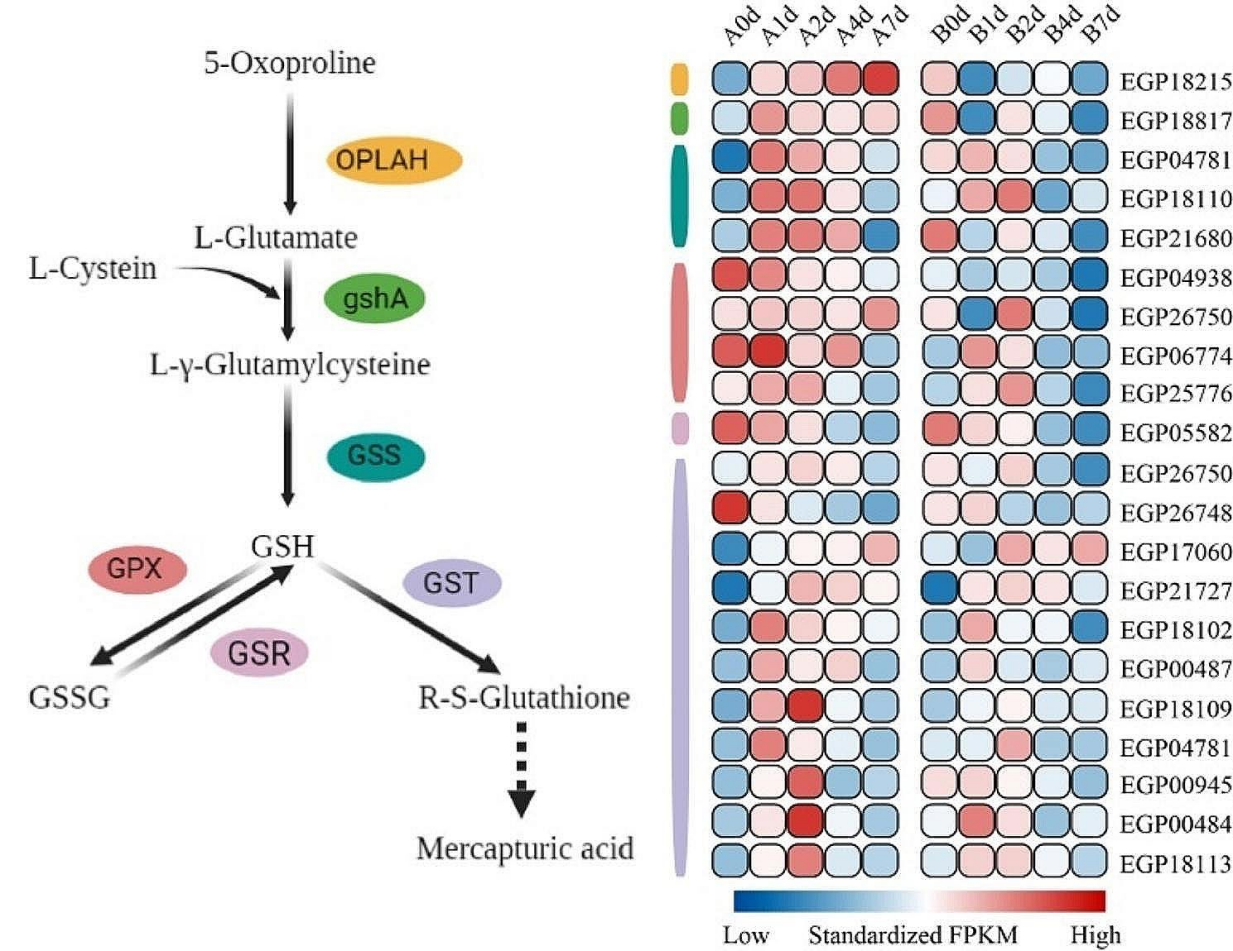
Metabolic pathways of glutathione and expression profile of related genes. GST, glutathione S-transferase; GSS, glutathione synthetase; GPX, glutathione peroxidase; GSR, glutathione reductase; gshA, glutamate–cysteine ligase; OPLAH, 5-oxoprolinase (ATP-hydrolysing)

Glutathione, a vital antioxidant component, serves as a crucial indicator of plant response to stress [30]. In this study, 21 DEGs were identified in the transcriptome participating in the glutathione metabolism pathway. 19 DEGs in “A” were significantly upregulated, under cold stress. As shown in Fig. 5, most genes encoding enzymes regulated by the glutathione biosynthesis pathway exhibited a similar expression pattern, with the highest expression in “A” and the lowest in “B”. The expression levels of glutathione upstream synthesis genes (*OPLAH, gshA*) in “A” significantly increased at 1 d under cold stress, while in “B”, they were significantly downregulated. The expression level of *GSS* in “A” significantly upregulated after 1 d and returned to baseline at 7 d, while the transcriptional abundance in “B” remained lower than that in “A”. The expression patterns of DEGs (*GPX*, *GSR*, and *GST*) downstream of glutathione metabolism were consistent between “A” and “B” at different time points, yet the transcriptional abundance of DEGs in variety “B” was consistently lower than that in variety “A” (Fig. 5). These results indicated that the glutathione metabolism pathway is an important metabolism in cold resistance “A” under cold stress, and the transcriptional abundance of various DEGs was significantly higher than that in “B” under cold stress.

**Fig. 6:**
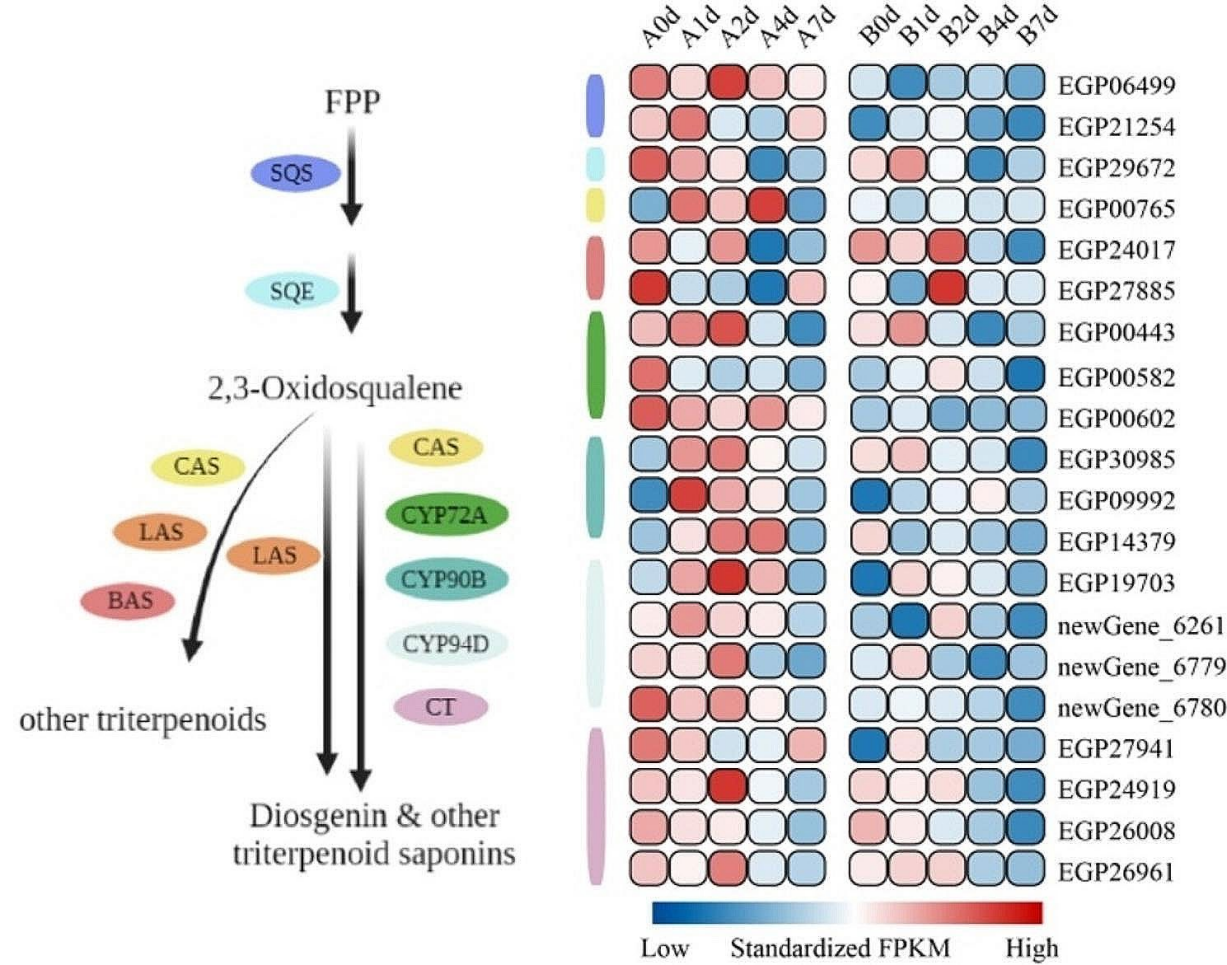
The metabolic pathways skeleton of terpenoids and the expression profile of related genes. SQS, squalene synthase; SQE, squalene epoxidase; CAS, cycloalkene synthase; LAS, lanosterol synthase; BAS, b-myristica synthetase ; cytochrome P450 CYP72A; cytochrome P450 CYP90B; cytochrome P450 CYP94D; CT, carboxytransferase

Terpene compounds exhibit antioxidant effects, effectively neutralizing free radicals and mitigating cellular damage caused by oxidative stress. KEGG enrichment analysis highlighted the specific enrichment of triterpene synthesis pathways in variety “A”. To delve into this, we analyzed the expression patterns of triterpene biosynthetic genes in both “A” and “B” under cold conditions. The identification and gene expression analysis of cycloalkene synthase (*CAS*), lanosterol synthase (*LAS*), b-myristica synthetase (*BAS*), *CYP72A*, *CYP90C* and *CYP94D* cytochrome P450 family members in the trunk and branch lines showed different expression patterns in different strains (Fig. 6). The DEGs genes of “A” were highly expressed in the early stage (0–2d) of cold stress and significantly downregulated by the 7th d. Notably, the transcriptional abundance of various DEGs involved in the triterpenoid metabolic pathway in “A” under cold stress was markedly higher than that in “B”. In contrast, our transcriptome data unveiled that most triterpenoid biosynthetic genes in “B” were repressed with prolonged exposure to cold stress.

**Fig. 7:**
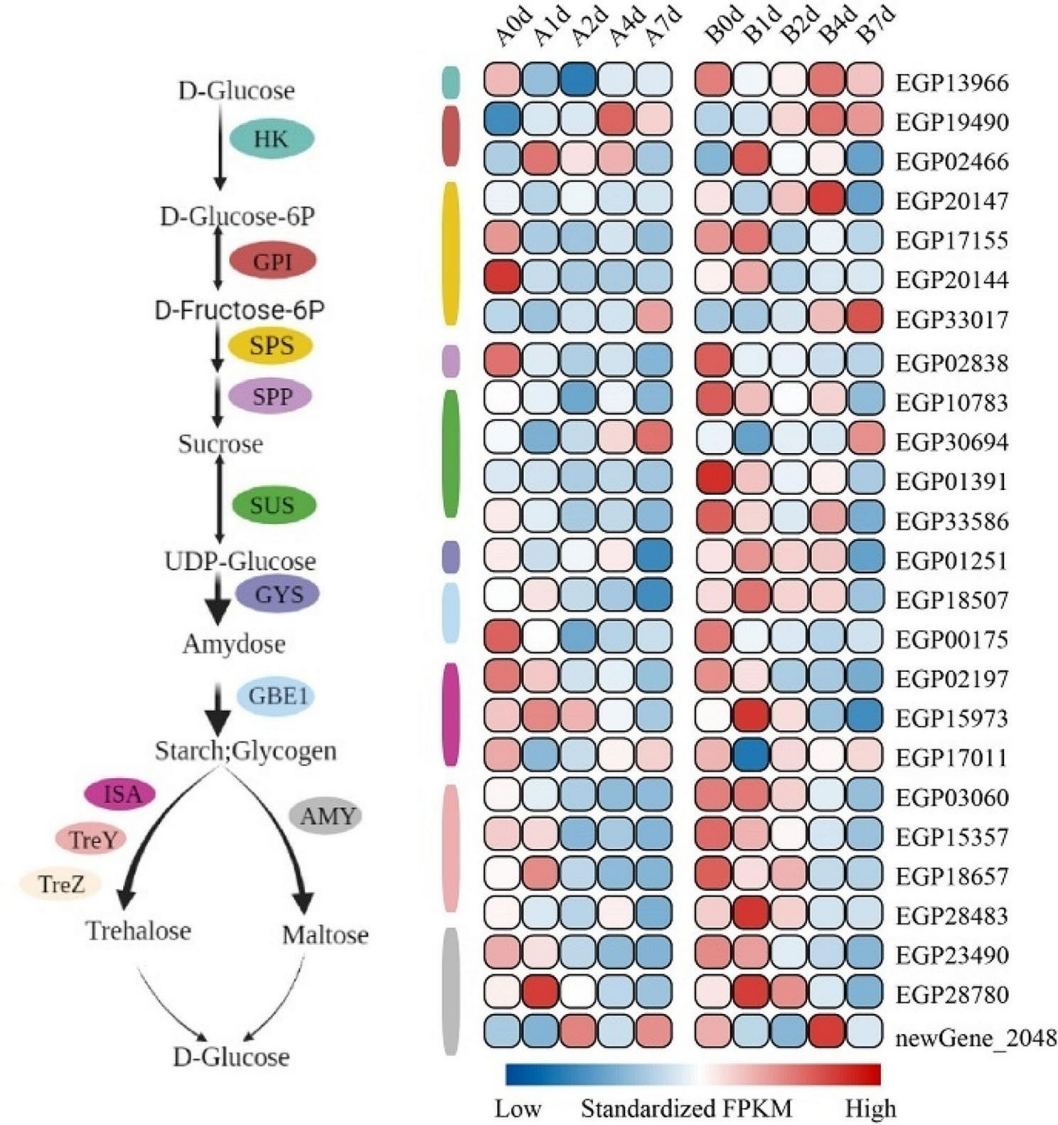
The biosynthetic skeleton of starch and sucrose and the expression profile of related genes. HK, hexokinase; GPI, glucose-6-phosphate isomerase; SPS, sucrose-phosphate synthase; SPP, sucrose-6-phosphatase; SUS, sucrose synthase; GYS, glycogen synthase; GBE1, 1,4-alpha-glucan branching enzyme; ISA, isoamylase; TreY, (1->4)-alpha-D-glucan 1-alpha-D-glucosylmutase; TreZ, maltooligosyltrehalose trehalohydrolase; AMY, alpha-amylase

Soluble sugar content serves as a pivotal physiological indicator, often reflecting a plant’s resistance under adverse conditions. To understand the impact of cold stress on the synthesis of soluble sugars in both “A” and “B”, we analyzed the expression patterns of genes in the core pathway of sugar synthesis under cold stress. We found that most of the transcripts related to the starch and sucrose pathway, including genes encoding isoamylase (*ISA*), (1->4)-alpha-D-glucan 1-alpha-D-glucosylmutase (*TreY*), maltooligosyltrehalose trehalohydrolase (*TreZ*), alpha-amylase (*AMY*) were more highly expressed in “B” than “A” (Fig. 7). Notably, most key starch and sucrose biosynthesis structural genes displayed lower expression levels on the 7th day. However, the *HK* gene showcased a consistent, higher expression level in the 4–7 day cold stress period of “B” (Fig. 7). *HK*, considered a key rate-limiting enzyme in EMP, displayed stable, higher expression levels in cold-stressed “B”, indicating that cold stress amplified the EMP metabolism of “B”, with its metabolic intensity surpassing that of “A”.

**Fig. 8:**
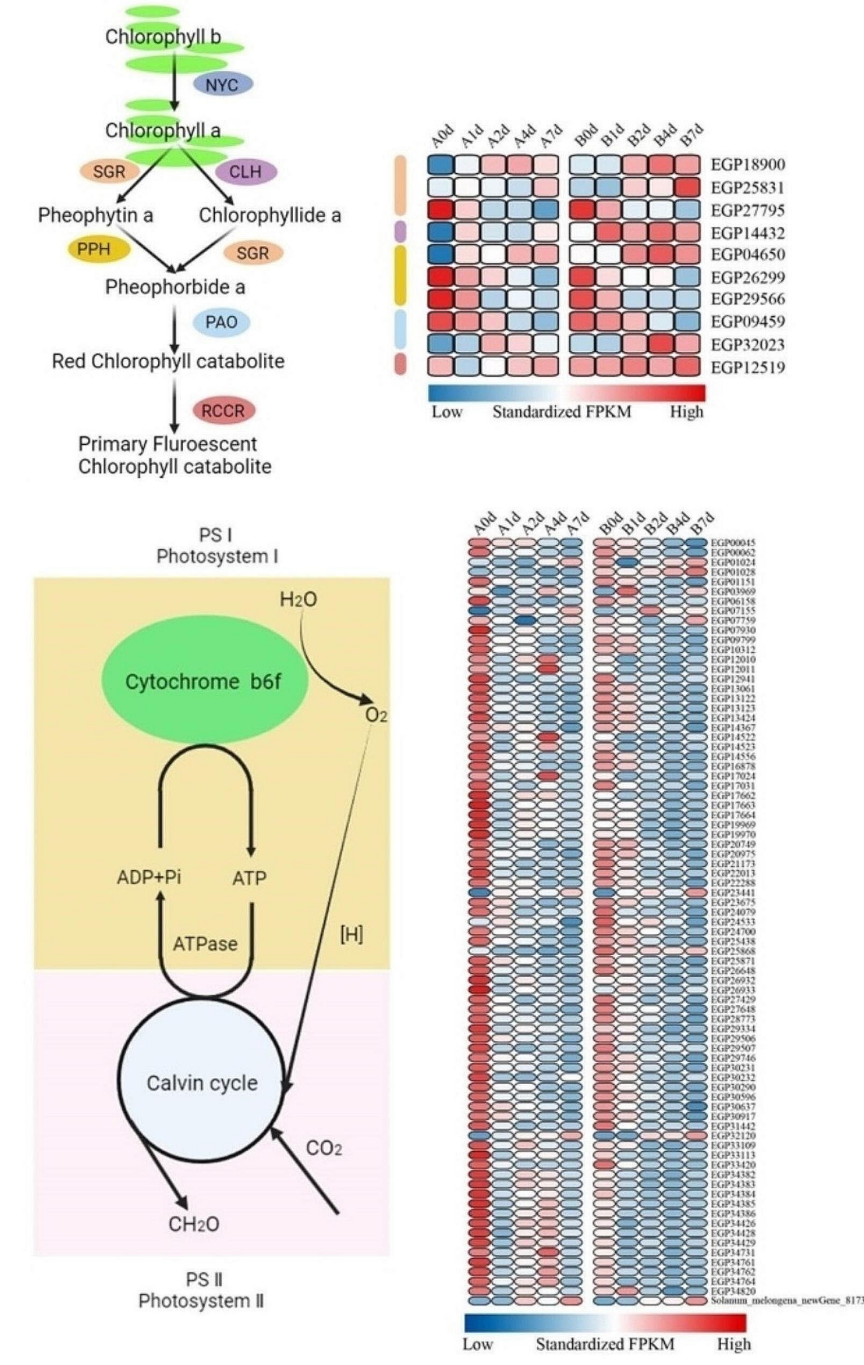
Expression profile of genes related to the metabolic pathway of photosynthesis. (**A**) Expression profile of backbone genes in chlorophyll degradation pathway. NYC, Non-yellow Coloring; SGR, Staygreen; CLH, Chlorophyllase; PPH, Pheophytinase; PAO, Pheophorbide a oxygenase; RCCR, Red chlorophyll catabolite reductase (**B**) Expression profiles of genes involved in photosystem I and photosystem II

When subjected to adverse stress, the wilting of plant leaves is often accompanied by the rapid breakdown of chlorophyll. Transcription data revealed 10 DEGs chlorophyll degrading genes, including 3 stage green (*SGR1*, *SGR2*, *SGR3*), 1 chlorophyllase gene (*CLH*), 3 phenolase genes (*PPH1*, *PPH2*, *PPH3*), 2 phenophoride an oxygenase genes (*PAO1*, *PAO2*), and 1 red chlorophyll catabolite reductase gene (*RCCR*). Among them, *SGR3*, *PPH2*, *PPH3*, and *PAO1* were downregulated after non cold stress treatment, but other chlorophyll degrading genes, especially *CLH*, *SGR1*, *SGR2*, *PPH1*, *PAO2*, and *RCCR* were upregulated (Fig. 8a). The upregulation of gene “B” during cold treatment was more pronounced compared to that of gene “A”. These results suggest that cold stress triggers the degradation of chlorophyll and activates the corresponding biochemical processes. The degradation of chlorophyll leaded to a weakening of photosynthesis, which inhibited plant growth and development. By KEGG enrichment analysis, we identified DEGs associated with photosynthesis to unravel the molecular mechanisms governing varied photosynthetic responses in the two varieties during cold stress treatment. The KEGG analysis pinpointed a total of 79 DEGs involved in the photosynthesis pathway, with 55 DEGs linked to Photosystem I and 24 DEGs related to Photosystem II (Fig. 8b, Table S3). Notably, 14 genes exhibited upregulation in the leaves of “A”. DEGs associated with various facets of photosynthesis, including light harvesting and electron transfer chains, were significantly enriched. Under cold stress, the expression of Photosystem II (PS II) genes (*PsbA*, *PsbC*, *PsbB*, *PsbK*, *PsbQ*, *PsbR*, *PsbY*, and *PsbW*) and Photosystem I (PS I) proteins (*PsaA*, *PsaB*, *PsaC*, and *PsaD*) genes was downregulated in both varieties. However, in comparison to the expression levels in “A”, the majority of DEGs in “B” exhibited lower expression, indicating a significant decrease in photosynthetic activity in “B”. This decrease in photosynthesis is closely linked to the variety’s tolerance to low temperatures.

### Transcription factor analysis and weighted correlation network analysis (WGCNA)

**Fig. 9:**
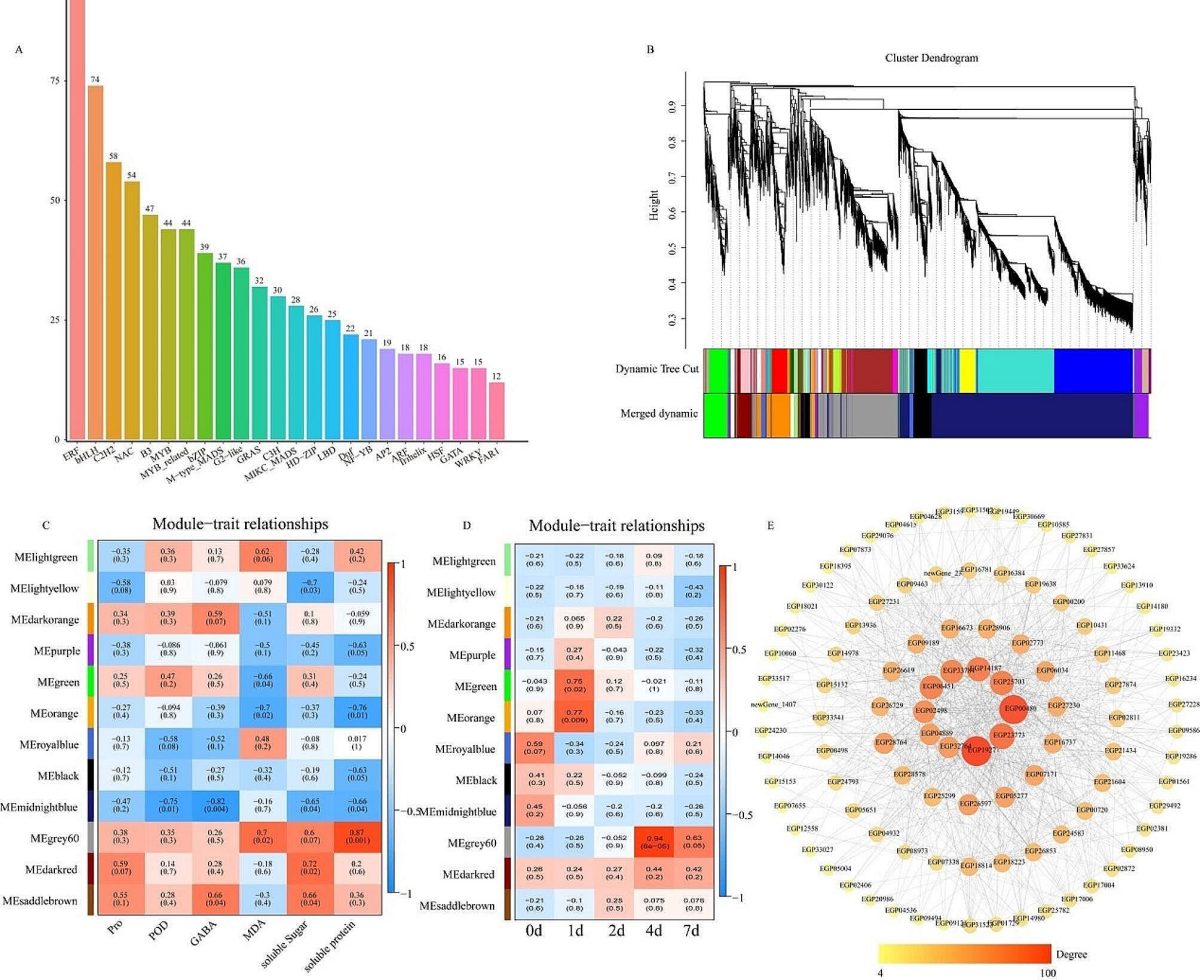
Transcription factors and Weighted Gene co-expression network analysis of cold response related genes in eggplant. (**A**) Soft threshold graph. The horizontal axis represents the soft threshold (β), the vertical axis represents the fitting index (left) and average connectivity (right) of the scale-free topology model. (**B**) Cluster tree diagram and module partitioning of genes. (**C**) Display the correlation between modules and traits (physiological indicators). (**D**) The correlation and characteristics (time) between modules. Each row corresponds to a module, and each module cell contains the corresponding correlation and p-value. (**E**) The co expression network of the top 150 core genes responsive to cold stress in the MEgrey 60 module. The node size represents connectivity

The fluctuations in gene expression levels are pivotal in regulating eggplants’ response to cold stress, with transcription factors (TFs) playing a crucial role in both biological and abiotic stress responses. A total of 823 hypothetical TFs belonging to 24 different families were identified, with the top 10 TF families illustrated in Fig. 9a. WGCNA was employed to further explore the relationship among key genes, stress time and physiological characteristics (genes filtered through FPKM < 1). WGCNA delineated highly correlated gene clusters termed modules, where genes within the same cluster exhibited strong correlations. A total of 12 modules were optimized and merged based on identified genes (Fig. 9b). WGCNA was used to further explore the relationship among key genes, stress time and physiological characteristics (genes filtered through FPKM < 1). WGCNA results showed that highly correlated gene cluster were defined as modules, and genes in the same cluster were highly correlated. The identified genes were optimized and merged into 12 modules (Fig. 9b). The correlation analysis of characteristic genes traits between each module and cold related physiological indicators showed that MEgray60 (*r* = 0.87, *p* < 0.05), MEdarkred (*r* = 0.72, *p* < 0.05), and MEsaddlebrown (*r* = 0.66, *p* < 0.05) were significantly positively correlated with physiological indicators, while MElightyellow (*r* = -0.70, *p* < 0.05), MEgreen (*r* = -0.66, *p* < 0.05), MEorange (*r* = -0.76, *p* < 0.05), and MEmidlightblue (*r* = -0.82, *p* < 0.05) were significantly negatively correlated with physiological indicators (Fig. 9c). Using the same method and setting parameters, MEgrey60 (*r* = 0.94, *p* < 0.05) emerged as having a robust correlation with cold stress duration and physiological indicators (Fig. 9c, d). This finding suggests that co-expressed genes within the MEgrey60 module are intricately linked to cold-related physiological indicators and the duration of cold stress, signifying their pivotal role in eggplant’s response to cold stress. Consequently, co-expressed genes within the MEgrey60 module were selected for further in-depth analysis and research.

The regulatory network comprising the top 150 hub genes, characterized by high connectivity, was visualized using Cytoscape software v3.9.1 (https://cytoscape.org/download. html) (Fig. 9e). In the MEgrey60 module, 10 central genes were identified based on specific criteria (kME ≥ 0.9) and edge weights ≥ 0.5. These genes were predominantly associated with transcriptional activation, plant hormone regulation, and redox homeostasis. Notably, *POD*_EGP13161, *PP2C*_EGP11066, *SnRK2*_EGP05474, *SnRK2*_EGP18530, *MDR1*_EGP04516, *DELLA*_EGP22417, *PRR7*_EGP18322, and transcription factors (C2H2_EGP19213, AP2/ERF_EGP27974, MYB_EGP33116, bHLH_EGP07396, bZIP_EGP13341, and ERF_EGP29469) were identified as central genes that may play crucial roles in the cold response of eggplants. Subsequently, a functional annotation analysis of other genes within the co-expression network revealed enrichment in eight main aspects: signal transduction, plant hormone regulation, biosynthesis and metabolism, transcription factors, cell structure, ion binding, catalytic and transport activities, and abiotic stress (Fig. S3).

Specifically, transcription factors C2H2_EGP19213, AP2/ERF_EGP27974, MYB_EGP33116 were co expressed with genes related to hormones (including auxin (*AUX1*_EGP18001, *IAA*_EGP14445, *ARF*_EGP31412), ABA (*PP2C*_EGP11066, *SnRK2*_EGP05474, *SnRK2*_EGP18530). MYB_EGP33116, bZIP_EGP13341, and ERF_EGP29469 related genes are co-expressed in redox homeostasis, including *POD*_EGP13161, *GST*_EGP00487, *GST*_EGP18109, *GR*_EGP00582, and *APX*_EGP30903. Finally, MYB_EGP33116, C2H2_EGP19213 and bHLH_EGP07396 were co expressed with genes related to soluble sugar synthesis genes (*SPS*_EGP10783, *SUS*_EGP10783, *GBE1*_EGP00175, and *UDPGA*_EGP04238) and genes related to cold stress signaling (*MPK*_EGP00891, *CAMAL*_EGP04153, and *CLR*_EGP10353). In conclusion, MYB was the core hub of the network, these results suggest that MYB_EGP33116 may be a central gene regulating plant hormone and TF, which cooperatively regulates redox homeostasis, MPK signaling pathway and soluble sugar biosynthesis, thus protecting eggplant seedlings from cold stress.

### QRT-PCR validation of RNA-seq data

To verify the accuracy of RNA-seq results, a total of 16 genes were randomly selected for qRT-PCR experiments, including EGP06774, EGP09992, EGP06499, EGP14379, EGP00263, EGP25367, EGP09657, EGP20989, EGP01302, EGP14445, EGP18001, EGP11066, and EGP18530. The gene expression levels of the two varieties at five time points were measured. Pearson correlation analysis showed that the multiple changes of qRT-PCR and RNA-seq were basically the same (Fig. S4), indicating the reliability of transcriptome sequencing.

## Discussion

When plants face low-temperature stress, a cascade of cellular physiological activities is triggered, initiating a series of cold stress response processes to cope with environmental challenges. To elucidate the mechanism of eggplant response to cold stress, the physiological and transcriptome response patterns between cold tolerant and cold sensitive varieties of eggplant were compared and analyzed. Firstly, the analysis of physiological data results shows that alterations in GABA, MDA, free proline, soluble protein, and soluble sugar content may be pivotal factors influencing the tolerance of eggplant species to low-temperature stress. Comparative analysis of transcriptome between “A” and “B” indicated that cold stress has a significant impact on the abundance of transcripts A and B, notable differences in the cold-responsive gene profiles between the two varieties are evident (Figs. 1 and 2). KEGG enrichment analysis shown that plant hormones and signal transduction systems, starch and sucrose metabolism, and diterpenoid and tetraterpene synthesis may be important roles in the response to cold stress in these eggplant species. In the subsequent sections, we delve into the interpretation of these crucial findings.

Abiotic stresses, such as cold stress, inflict damage on the cell membrane by inducing oxidative and lipid peroxidation processes [31–33]. In this study, a significant increase in the MDA content in both “A” and “B” species under low temperatures indicates heightened lipid peroxidation and membrane injury. Under oxidative conditions, the accumulation of uncontrolled free radicals prompts plants to employ enzymatic and nonenzymatic antioxidants to mitigate oxidative stress and maintain cellular homeostasis. POD, a key enzyme in the plant’s enzymatic defense system during stress, collaborates with SOD and CAT to eliminate excess free radicals and enhance the plant’s stress resistance [34]. Previous research has shown increased POD activity in cold-tolerant banana varieties under cold stress, contrasting with a significant decrease in cold-sensitive varieties [35]. Similarly, *Zanthoxylum bungeanum* exhibited enhanced POD activity after cold stress, crucial in reducing ROS accumulation [36]. In our study, POD activity increased with prolonged cold stress, and the activity of POD in “A” was notably higher than in “B” from 0 to 4 days. These results suggest that a robust antioxidant system enhances the ROS clearance efficiency of “A”. Moreover, our research revealed a positive correlation between the expression level of POD coding genes and POD activity (*p* < 0.05), indicating that the upregulation of these genes contributes to increased POD enzyme activity. In summary, these findings demonstrate that lipid peroxidation reduction in “A” mitigates cell damage. Compared to “B”, “A” exhibits a more robust antioxidant system, effectively eliminating ROS and adapting to cold environments.

Under low-temperature stress, plants synthesize a substantial amount of permeable substances to reduce the osmotic potential of cells and bolster the water retention capacity of plants [4, 5]. In our study, the content of soluble proteins, soluble sugars, free proline, and GABA increased with the prolonged duration of cold stress. Throughout most of the cold stress periods, “A” exhibited higher soluble protein content than “B”, whereas “B” demonstrated higher levels of soluble sugars, free proline, and GABA. This discrepancy helps maintain a greater cell osmotic potential in “B”, facilitating water absorption by plant roots and retaining water in cells. Proline is considered the primary osmotic agent in higher plants, preventing water loss, and its accumulation is a common physiological response to cold stress. Enhanced cold resistance in winter rapeseed under cold stress is associated with proline accumulation. Cold stress promotes proline accumulation in *Brassica rapa* L. ssp. Pekinensis seedlings, with proline content positively correlated with the degree of cold stress [20]. Conversely, research on pepper under cold stress indicates that the proline content in the cold-tolerant variety “FG” is higher during early and late stress stages compared to the cold-sensitive variety “FX” [36]. Further investigation is needed to explore the relationship between cold stress and proline changes in the plant body. Based on the results of this study, we posit that eggplants employ similar strategies to cope with low temperatures, with the antioxidant system and osmotic regulatory substances playing pivotal roles in managing low-temperature stress.

After prolonged exposure to cold stress, plants develop a strategy that involves coordinating cold and hormone signaling pathways, along with the MAPK cascade, to effectively cope with the challenges posed by low temperatures [37–41]. The RNA-seq analysis unveiled the regulation of numerous genes associated with abscisic acid, auxin, and jasmonic acid under cold stress in eggplants. Notably, three hormone-related GO terms—“response to abscisic acid stimulus”, “response to auxin stimulus”, and “response to jasmonic acid stimulus”—were significantly enriched among the differentially expressed genes in both “A” and “B” under cold stress (Fig. 3c,d). The significance of ABA core components in responding to low-temperature stress has been validated across diverse plant species. For instance, heterologous overexpression of the SnRK2 protein kinase gene from *Agropyron cristatum* (*AcSnRK2.11*) has demonstrated the ability to stimulate the growth of transgenic plants under normal conditions and enhance the tolerance of transgenic yeast and tobacco to cold stress [42]. Heterologous overexpression of a common wheat gene, *TaSnRK2.4*, has been shown to improve cold resistance by fostering the accumulation of ABA and augmenting ABA signaling in *Arabidopsis* [43]. The co-expression network analysis in this study identified the core components of the ABA signaling pathway, including *PP2C* and *SnRK2*, along with the downstream transcription factor ABF. These identified genes collectively form a comprehensive ABA pathway. Notably, the expression levels of three *SnRK2* coding genes and one ABF gene exhibited a significant increase after 4 days in the “A” group compared to the “B” group. This overall upregulation suggests that higher ABA accumulation may contribute to the stronger cold resistance observed in variety “A”. Auxin, a type of β-indoleacetic acid hormone, plays a crucial role in regulating plant cell division, differentiation, and responses to both abiotic and biotic stresses. In recent years, there has been growing research interest in understanding the impact of auxin on cold tolerance. In our study, we identified the auxin influx vector *AUX1* gene, crucial for auxin signal transduction, along with the *IAA* gene and downstream transcription activator ARF in the co-expression network. The expression levels of *AUX1* (EGP18001) and *ARF* (EGP02058) in the “A” group increased, promoting the accumulation of auxin and enhancing cold resistance. These findings underscore the significant role of plant hormones in eggplant cold stress responses.

The MAPK (mitogen-activated protein kinase) signaling pathway is a crucial intracellular signaling cascade that regulates various cellular responses, including responses to environmental stresses such as cold. MAPKs are enzymes that function by phosphorylating other proteins, thereby activating or inhibiting their activities [44, 45]. We identified two *MAPK* genes (EGP00891, EGP30996) with high connectivity in the co-expression network. However, the role of *MAPK* in the cold stress response is not yet well-understood. In the cold tolerance eggplants, the upregulation of two genes encoding MAPKs suggests that these genes are actively involved in the plant’s response to cold stress. Upregulation typically indicates that the genes are being expressed at higher levels, which may lead to increased production of the corresponding MAPK proteins. These proteins, in turn, may phosphorylate and activate other proteins involved in cold tolerance mechanisms, such as transcription factors or other kinases. Moreover, these two *MAPK* genes in variety “A” were upregulated after 4 d of cold treatment, suggesting a higher efficiency of MAPK signal transduction in “A” compared to “B”. This may lead to the activation of multiple cold reaction pathways in “A”. On the other hand, the downregulation of these two genes in cold-sensitive eggplants suggests that the MAPK signaling pathway may not be fully functional or effective in these varieties. This downregulation may lead to reduced production of the MAPK proteins, which could impair the plant’s ability to mount an effective response to cold stress. In summary, under low-temperature stress, the differential gene expression may impact the biosynthesis of ABA and IAA, leading to an increase in the content of these hormones in eggplant seedlings. This overall hormonal modulation may induce cell signaling pathways, particularly MAPK, associated with stress tolerance, ultimately mitigating the adverse effects of stress on growth by elevating the levels of plant hormones such as ABA and auxin.

Transcription factors play a crucial role in regulating gene expression by specifically binding to the upstream promoter sequences of functional genes, including secondary metabolic pathways, defense responses, as well as growth and development regulation [14–16]. By orchestrating these interactions with specific genes, transcription factors contribute significantly to the overall phenotype and adaptability of plants in response to various internal and external stimuli. Many plant species have identified and analyzed several cold stress-responsive TF families [46]. For instance, the heterologous overexpression of soybean *GmWRKY21* has been demonstrated to enhance *Arabidopsis* resistance to cold stress [47]. Another study showed that the heterologous overexpression of the WRKY transcription factor *PmWRKY57* in plum blossoms significantly enhanced cold resistance in *Arabidopsis*. *CaNAC2* was strongly induced by low temperature and gene silencing techniques induced by viruses were used to inhibit the expression of *CaNAC2* pepper (*Capsicum annuum* L.) seedlings, increasing their sensitivity to low temperatures [48]. Additionally, *GmNAC20* was identified as a positive regulator of salt and freezing resistance in transgenic Arabidopsis plants [49]. In recent studies, multiple cold-responsive transcription factors have been identified in eggplants [28, 29]. The correlation of five key transcription factors (MYB, AP2/ERF, bZIP, bHLH, C2H2) with cold stress in eggplants highlights their potential importance in mediating plant responses to environmental challenges. While previous studies have identified these transcription factors in relation to cold stress in various plant species, the specific regulatory mechanisms in eggplants require further elucidation. In particular, the role of MYB transcription factors in eggplant cold stress response mechanisms remains unexplored in the current literature. Investigating the involvement of MYB transcription factors in regulating cold stress responses in eggplants could offer valuable insights into novel regulatory pathways and contribute to a more comprehensive understanding of plant stress responses.

Some unique metabolites in plants are often associated with defense responses because they activate defense related genes. The synthesis and accumulation of secondary metabolites in plants in response to stress play a crucial role in enhancing the plant’s resistance mechanisms. Studies had shown that under stress conditions such as low temperatures, plants can trigger the accumulation of compounds like flavonoids. Flavonoids are known for their antioxidant properties and had been associated with improving plant stress resistance by scavenging free radicals and protecting plant cells from oxidative damage [50]. Sucrose can elevate metabolite levels and enhance specific enzyme activity through various pathways, thereby bolstering plant stress resistance. For instance, Yuan et al. observed a significant upregulation of genes encoding terpenoid synthase/trehalose 6-phosphate phosphatase (*TPS/TPP*) and trehalose 6-phosphatase (*TREH*) in the drought-tolerant millet variety DT43 under drought and melatonin treatment, leading to increased trehalose accumulation and improved drought resistance [51]. Yang et al. conducted KEGG analysis on cold-tolerant and cold-sensitive coconut varieties subjected to low-temperature stress, revealing a substantial enrichment of differentially expressed proteins in starch and sucrose metabolism pathways [52]. In upland cotton of the KN27-3 type, genes encoding GDP-neneneba mannose, trehalose, and raffinose were highly expressed under low-temperature stress, promoting soluble sugar accumulation and protecting cotton from oxidative damage [53]. In the context of cold-tolerant eggplant plants, our study identified significantly higher expression levels of sucrose phosphate synthase (*SPS*), glycolysis, and UDP glucosyltransferase related to sucrose metabolism compared to cold-sensitive eggplant plants. This heightened expression likely maintained a more robust gene expression profile in cold-tolerant eggplant plants, enhancing resistance to the permeation environment imbalance induced by cold stress. Terpenoids, a subclass of terpenes, are a diverse group of organic compounds found in plants that exhibit a wide range of biological activities, including antioxidant activity and influencing plant growth, development, and stress response. Research by Li et al. had demonstrated that low temperatures can induce the accumulation of volatile orange tertiary alcohol and glycoside precursors in tea plants, enhancing their cold resistance [54]. Similarly, Li et al. observed an increase in the content of terpenoids in sandalwood leaves under cold stress, contributing to enhanced stress resistance [55]. In this study, the backbone genes (*SQS*, *CAS*, *CYP72A*, and *CYP94D*) related to terpenoid synthesis was identified in the co-expression network, and the expression levels of these genes were higher in “A” compared to “B.” By identifying and characterizing the genes involved in terpenoid biosynthesis and understanding how their expression is modulated in response to cold stress, researchers can gain insights into the molecular mechanisms underlying cold tolerance in plants. This knowledge can be leveraged to develop new strategies for enhancing cold tolerance in crop plants, improving their performance under adverse environmental conditions.

**Fig. 10:**
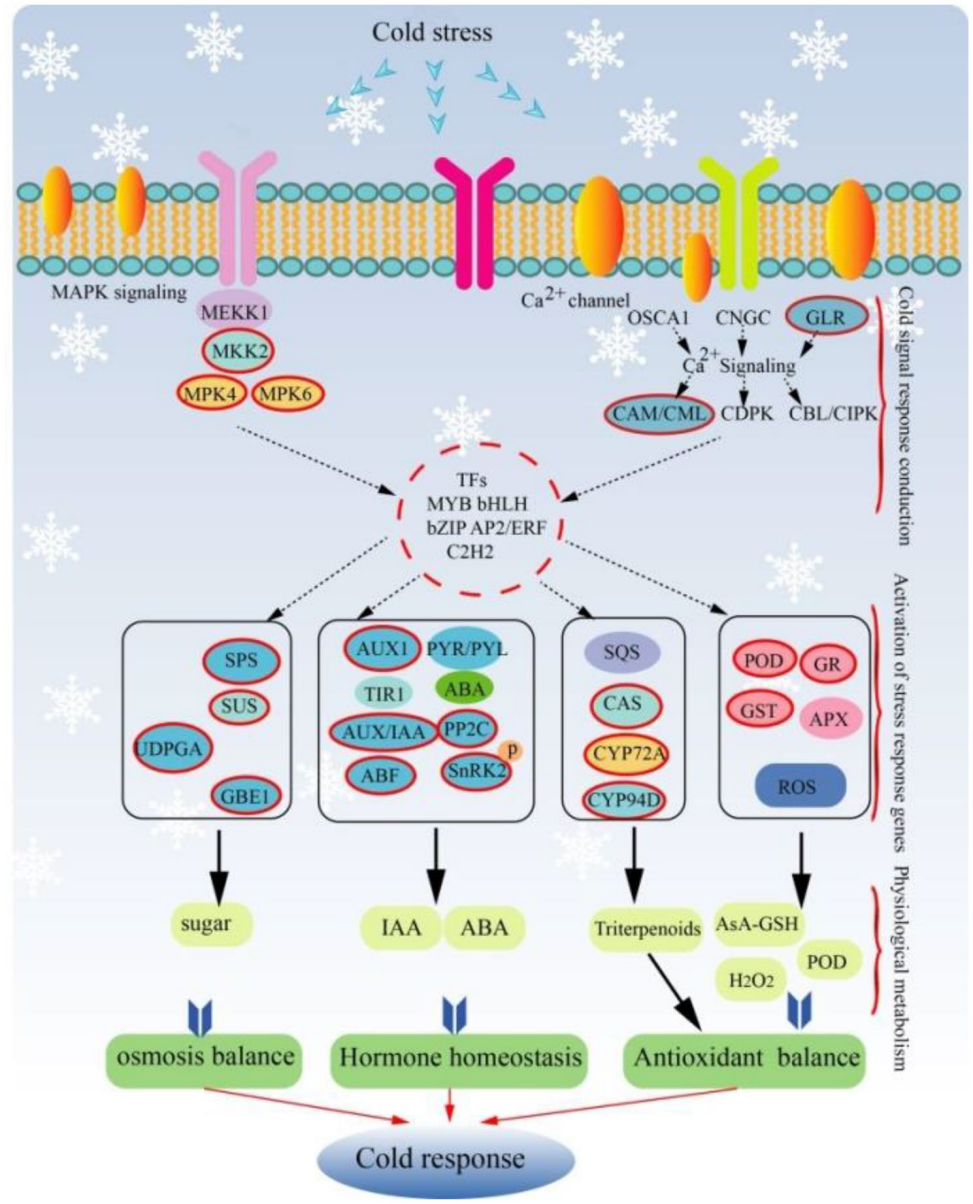
Hypothetical modules and cold response pathways of eggplant. The red box represents the cold response factor identified from the co-expression network

These findings present a more comprehensive research approach to investigating plant stress resistance, spanning physiological, transcriptional, and protein levels. This integrated approach better elucidates the cold response mechanism in plants. Using the identified cold response factors, we established a comprehensive model illustrating the eggplant cold response mechanism, represented by a complex co-expression network (Fig. 10). This model holds the potential to uncover the intricate response network pathways in eggplants under cold stress, offering valuable insights for studying the cold tolerance of other plant species.

## Conclusions

The physiological and molecular response mechanisms to cold stress in eggplants are intricate, involving various factors. Physiological index detection and chemometrics analysis revealed distinct regulation factors for the cold-tolerant variety “A” and the cold-sensitive variety “B”. In “A”, POD and soluble proteins were pivotal for a more responsive reaction to cold stress, while osmotic regulators in “B” played a crucial role in the later stages of cold stress treatment. Genes associated with glutathione, terpenoids, and starch and sucrose metabolism pathways were identified as key players in regulating the cold response. The construction of a co-expression network, integrating physiological and transcriptome data, illustrated that eggplants employ signal transduction, plant hormones, transcription factors, membrane transporters, and cell structure in response to cold stress. Core genes like *POD*, *PP2C*, *SnRK2*, *MYB*, *ERD7*, *DELLA*, *PRR7*, and transcription factors within the co-expression network played central roles in eggplant cold response. The findings from this study serve as a valuable reference for understanding the cold response mechanisms of plants, including eggplants, under low-temperature stress.

## Materials and methods

### Plant materials and cold treatment

Two *S. melongena* varieties, “E7134” (“A”, a cold-tolerant variety) and “E7145” (“B”, a cold-sensitive variety) were selected for cold treatment. The seeds of two varieties were manually harvested at the natural maturation period, respectively. All seedlings were grown in a greenhouse at 26 ± 1 °C and 16/8 h (light/dark) in the agricultural science of sichuan province (Chengdu, China) before experiment. For cold treatment, eggplant seedlings with 4 to 5 leaf sizes were transferred to a growth chamber, where the daily photoperiod was 16 h/8 h (light/dark), the temperature was 5 °C/10°C (day/night). A total of 30 leaves samples from two *S. melongena* varieties seedling were collected after 0, 1, 2, 4, and 7 d with 3 biological replicates obtained at each experimental time point, immediately frozen in liquid nitrogen and stored at -80 °C for further studies.

### Determinations of leaf physiological indices under 5 ◦C cold stress

To ensure sample integrity and prevent enzyme inactivation, 0.1 g of leaf tissue, previously frozen in liquid nitrogen, was weighed. Subsequently, 1 mL of extraction solution was added, and homogenization was carried out in an ice bath. After centrifugation at 8000 rpm for 10 min at 4 °C, the supernatant was carefully collected and kept on ice for further measurements [56, 57]. The activity of POD and the content of various metabolites (MDA, free proline, soluble protein, GABA, and soluble sugar) were quantified in 1 mL of leaf sample supernatant using specific kits, following the manufacturer’s instructions (Solarbio, Beijing, China). Results were expressed as mean ± standard deviation. Statistical analysis was conducted using GraphPad Prism 7. Differential analysis of metabolites between the two varieties was performed using PCA and OPLS-DA [58].

### RNA extraction, library preparation, RNA-Seq, and sequence assembly

For RNA sample preparation, 1 µg of RNA per sample served as input material. The concentration and purity of RNA were determined using NanoDrop 2000 (Thermo Fisher Scientific, Wilmington, DE, USA), while RNA integrity was assessed through the RNA Nano 6000 Assay Kit on the Agilent Bioanalyzer 2100 system (Agilent Technologies, CA, USA). Sequencing libraries were constructed using the NEBNext UltraTM RNA Library Prep Kit for Illumina (NEB, USA), following the manufacturer’s guidelines. Index codes were added to attribute sequences to each sample. In brief, mRNA was isolated from total RNA using poly-T oligo-attached magnetic beads. Fragmentation was achieved using divalent cations at an elevated temperature in NEBNext First Strand Synthesis Reaction Buffer (5X). First-strand cDNA synthesis was performed using a random hexamer primer and M-MuLV Reverse Transcriptase. Subsequent second-strand cDNA synthesis was carried out using DNA Polymerase I and RNase H. Remaining overhangs were converted into blunt ends via exonuclease/polymerase activities. After adenylation of 3’ ends of DNA fragments, NEBNext Adaptor with a hairpin loop structure was ligated for hybridization. To select cDNA fragments of approximately 240 bp in length, library fragments were purified using the AMPure XP system (Beckman Coulter, Beverly, USA). Subsequently, 3 µl USER Enzyme (NEB, USA) was applied with size-selected, adaptor-ligated cDNA at 37 °C for 15 min, followed by 5 min at 95 °C before PCR. PCR was executed with Phusion High-Fidelity DNA polymerase, Universal PCR primers, and Index (X) Primer. Finally, PCR products were purified (AMPure XP system), and library quality was assessed using the Agilent Bioanalyzer 2100 system. Clean data (clean reads) were obtained by eliminating reads containing adapters, reads containing poly-N, and low-quality reads from the raw data. Differential expression analysis of two samples was carried out using edgeR [59], with the threshold for significantly differential expression set at FDR < 0.01 & Fold Change ≥ 2.

### Co-expression network analysis and hub gene identification

Gene co-expression network analysis was performed using the Weighted Gene Co-Expression Network Analysis (WGCNA) package (v1.72-1) in R [60]. The analysis utilized RNA-Seq data from 30 samples (two varieties × five time points × three replicates). The key parameters included mean FPKM ≥ 1, a similarity threshold of 0.25 for control module fusion, and a minimum of 30 genes in each module. WGCNA divided genes with different expression levels into 12 modules, with distinct colors representing each module. The correlation between each module and cold stress duration, as well as the correlation with physiological indicator data, was calculated. DEGs were assigned to different modules using the Dynamic Tree Cut algorithm [61]. The node size was adjusted based on the number of genes linked to a specific gene. Node genes from modules with kME > 0.9, ranked by kME to the top 150, were selected as hub genes representing the overall expression trend of the respective module. Connectivity, defined as the sum of weights from all edges of a node, was used to assess the node’s importance [62, 63].

### Real-time quantitative PCR validation of DEG results

Total RNA was extracted from leaves treated with cold stress using the TRIzol reagent (Invitrogen, USA) [64]. Reverse transcription used RNA as a template was performed with the PrimeScript TM RT Reagent Kit with gDNA Eraser (TaKaRa, Japan). The specific primers used in this study were designed via Primer 5 v5.5.0 with RefSeq and are listed in Table S4, the expression of the β-tubulin gene was used as an internal reference. Quantitative real-time PCR (qRT-PCR) was carried out by a CFX96 Touch Real-Time PCR System (Bio-Rad, USA) with SYBR Premix ExTaqTM (Takara) using TB GreenTM Premix Ex TaqTM II (TaKaRa). Three independent biological and technical replicates of each sample were subjected to RT-qPCR analysis. The 2^−∆∆CT^analysis method was utilized to calculate relative expression levels. Subsequently, Pearson’s correlation analysis between the data obtained by RNA-seq and qRT-PCR was performed following Guo et al. [65] and the results were imported to the TBtools [66] to visualize the heat map of expression levels.

### Electronic supplementary material

Below is the link to the electronic supplementary material.

Supplementary Material 1

## Data Availability

The datasets used and/or analyzed during the current study are availablein the supplementary file, and additional datasets can be provided by the corresponding author on request. The raw sequence data reported in this paper have been deposited in the Genome Sequence Archive (Genomics, Proteomics & Bioinformatics 2021) in National Genomics Data Center (Nucleic Acids Res 2022), China National Center for Bioinformation / Beijing Institute of Genomics, Chinese Academy of Sciences (GSA: CRA013268) that are publicly accessible at https://ngdc.cncb.ac.cn/gsa. Not applicable.

## Abbreviations

*DEG*: Differentially expressed gene
*POD*: peroxidase
*OPLS-DA*: orthogonal partial least squares discriminant analysis
*MAPK*: mitogen-activated protein kinase
*WGCNA*: Weighted gene co-expression network analysis
*ROS*: reactive oxygen species
*ABA*: abscisic acid
*MeJA*: methyl jasmonate
*CAT*: catalase
*SOD*: superoxide dismutase
*CBFs*: C receptor binding factors
*ICEs*: inducers of CBF expression genes
*COR*: cold regulated
*MDA*: malondialdehyde
*GABA*: γ-aminobutyric acid
*NR*: Nonredundant
*GO*: Gene Ontology
*KEGG*: Kyoto Encyclopedia of Genes and Genomes
*NOG*: Non supervised Orthologous Groups
*GST*: glutathione S-transferase
*GSS*: glutathione synthetase
*GPX*: glutathione peroxidase
*GSR*: glutathione reductase
*gshA*: glutamate–cysteine ligase
*OPLAH*: 5-oxoprolinase (ATP-hydrolysing)
*SQS*: squalene synthase
*SQE*: squalene epoxidase
*CAS*: cycloalkene synthase
*LAS*: lanosterol synthase
*BAS*: b-myristica synthetase
*CT*: carboxytransferase
*HK*: hexokinase
*GPI*: glucose-6-phosphate isomerase
*SPS*: sucrose-phosphate synthase
*SPP*: sucrose-6-phosphatase
*SUS*: sucrose synthase
*GYS*: glycogen synthase
*GBE1*: 1,4-alpha-glucan branching enzyme
*ISA*: isoamylase
*TreY*: (1->4)-alpha-D-glucan 1-alpha-D-glucosylmutase
*TreZ*: maltooligosyltrehalose trehalohydrolase
*AMY*: alpha-amylase
*NYC*: Non-yellow Coloring
*SGR*: Staygreen
*CLH*: Chlorophyllase
*PPH*: Pheophytinase
*PAO*: Pheophorbide a oxygenase
*RCCR*: Red chlorophyll catabolite reductase
