# Supplementary material for: Comparative physiology and transcriptome response patterns in cold-tolerant and cold-sensitive varieties of Solanum melongena
Source: BMC Plant Biol. 2024 Apr 9;24:256. doi: 10.1186/s12870-024-04922-y (PMC11003173; doi:10.1186/s12870-024-04922-y)
Supplement: Supplementary file 1 — Supplementary Material 1 [file 12870_2024_4922_MOESM1_ESM.docx]

Table S1 Statistical results of sequencing data.

| Samples | Clean reads | Clean bases | GC Content | %≥Q30 |
| --- | --- | --- | --- | --- |
| A0d1 | 33,073,170 | 9,883,172,390 | 43.72% | 94.98% |
| A0d2 | 37,216,971 | 11,117,525,770 | 43.71% | 95.71% |
| A0d3 | 32,461,399 | 9,701,942,792 | 43.97% | 94.88% |
| A1d1 | 29,550,808 | 8,837,006,770 | 42.82% | 94.21% |
| A1d2 | 28,812,052 | 8,613,206,350 | 42.94% | 94.43% |
| A1d3 | 32,191,886 | 9,626,441,124 | 42.81% | 94.43% |
| A2d1 | 33,145,959 | 9,903,233,376 | 42.91% | 93.03% |
| A2d2 | 28,735,259 | 8,592,807,638 | 43.14% | 95.27% |
| A2d3 | 32,491,088 | 9,717,487,510 | 42.90% | 94.63% |
| A4d1 | 26,724,286 | 7,986,258,726 | 43.23% | 94.37% |
| A4d2 | 25,277,715 | 7,558,136,692 | 43.12% | 93.94% |
| A4d3 | 35,460,040 | 10,592,260,696 | 42.88% | 95.17% |
| A7d1 | 34,781,504 | 10,404,034,988 | 42.98% | 94.54% |
| A7d2 | 33,443,226 | 9,987,645,376 | 43.06% | 94.55% |
| A7d3 | 36,337,205 | 10,862,515,338 | 42.95% | 94.95% |
| B0d1 | 32,993,164 | 9,860,213,888 | 43.52% | 94.75% |
| B0d2 | 30,766,838 | 9,191,178,498 | 43.36% | 95.46% |
| B0d3 | 31,075,466 | 9,294,896,318 | 43.33% | 94.22% |
| B1d1 | 35,705,426 | 10,676,871,728 | 42.74% | 94.95% |
| B1d2 | 29,524,110 | 8,824,117,552 | 42.65% | 94.31% |
| B1d3 | 33,706,092 | 10,081,290,046 | 42.45% | 94.72% |
| B2d1 | 35,713,581 | 10,664,075,972 | 42.88% | 94.93% |
| B2d2 | 36,234,688 | 10,823,355,822 | 42.92% | 95.18% |
| B2d3 | 34,218,672 | 10,228,298,136 | 42.82% | 94.38% |
| B4d1 | 32,056,351 | 9,584,478,430 | 42.85% | 94.64% |
| B4d2 | 32,641,719 | 9,757,621,490 | 42.80% | 93.61% |
| B4d3 | 34,889,888 | 10,424,533,552 | 42.68% | 95.09% |
| B7d1 | 31,501,126 | 9,411,638,362 | 43.01% | 94.27% |
| B7d2 | 32,125,277 | 9,605,748,454 | 42.76% | 94.47% |
| B7d3 | 34,642,904 | 10,355,561,074 | 42.99% | 94.82% |

Table S2 Statistical analysis of gene function annotation results.

| Annotated databases | New Gene Number |
| --- | --- |
| COG | 8,993 |
| GO | 25,135 |
| KEGG | 21,137 |
| KOG | 15,986 |
| Pfam | 23,541 |
| Swiss-Prot | 20,270 |
| eggNOG | 27,095 |
| nr | 32,959 |
| All | 175,116 |


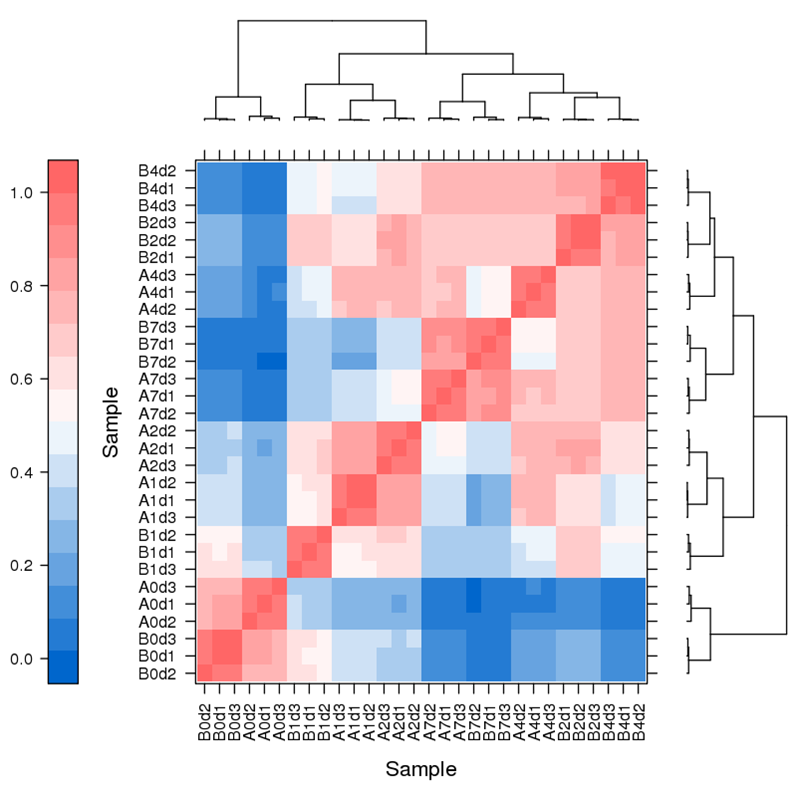


Fig. S1 Heat map of correlation between expression levels of pairwise samples.


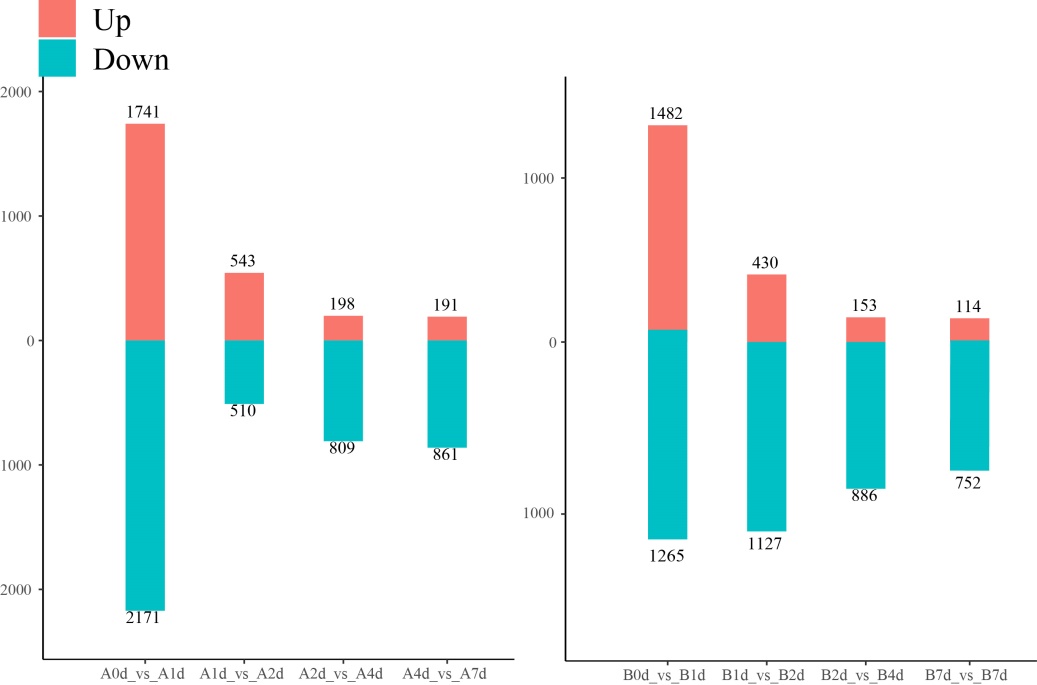


Fig. S2 Analysis of differentially expressed genes at different time points within the same variety.


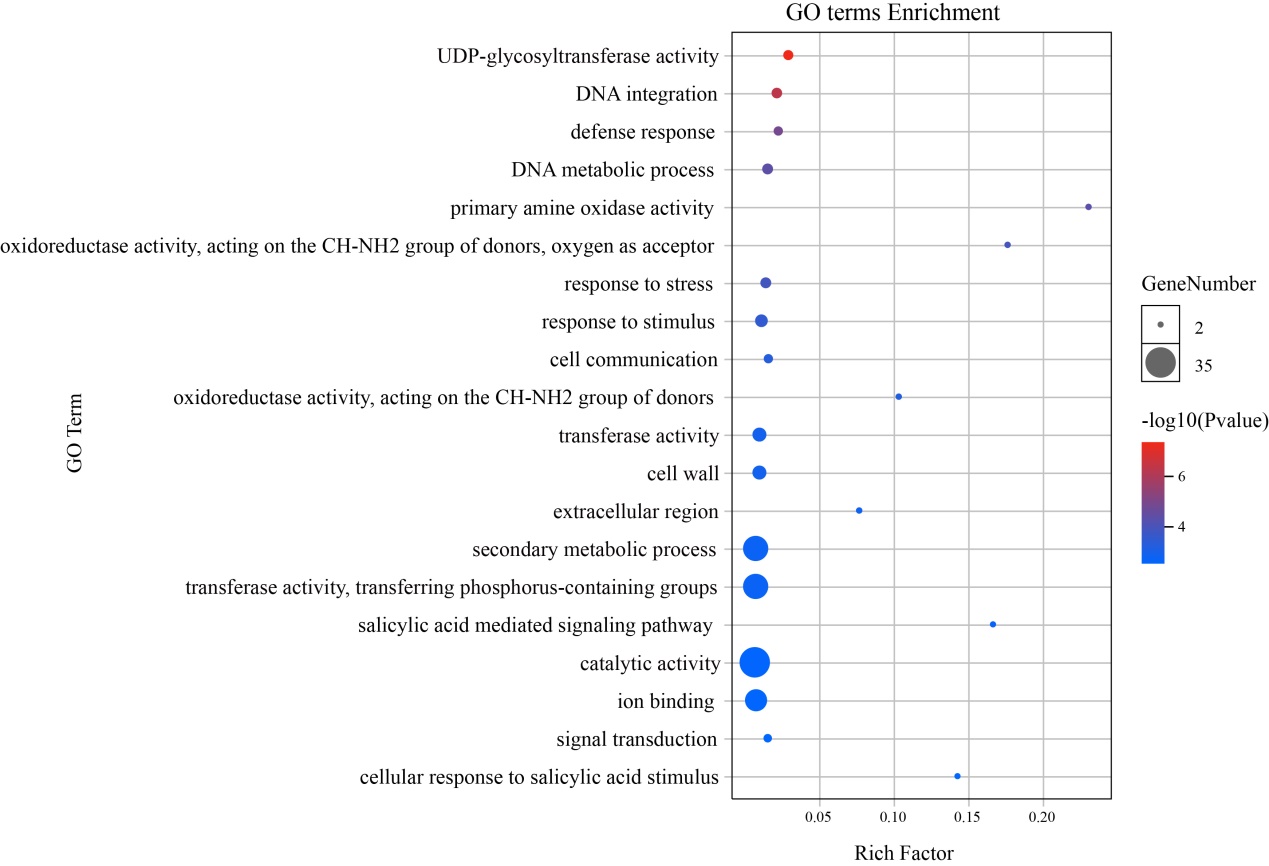


Fig. S3 GO enrichment analysis of the top 150 genes in the MEgrey60 co-expression network

Table S3 Annotated information of genes related to photosynthesis.

| ID | Database_ID | Annotation |
| --- | --- | --- |
| EGP00045 | NP_001275173.1 | photosystem II 10 kDa polypeptide, chloroplastic [Solanum tuberosum] |
| EGP00062 | XP_015082738.1 | photosystem I reaction center subunit V, chloroplastic [Solanum pennellii] |
| EGP01024 | XP_006344529.1 | PREDICTED: ferredoxin--NADP reductase, root-type isozyme, chloroplastic isoform X1 [Solanum tuberosum] |
| EGP01028 | XP_006344529.1 | PREDICTED: ferredoxin--NADP reductase, root-type isozyme, chloroplastic isoform X1 [Solanum tuberosum] |
| EGP01056 | XP_015162905.1 | PREDICTED: ferredoxin--NADP reductase, root-type isozyme, chloroplastic isoform X2 [Solanum tuberosum] |
| EGP01151 | TMW85415.1 | hypothetical protein EJD97_023200 [Solanum chilense] |
| EGP03359 | AWI73286.1 | photosystem II CP47 protein [Willughbeia edulis] |
| EGP03360 | EXB38499.1 | Photosystem II CP47 chlorophyll apoprotein [Morus notabilis] |
| EGP03969 | QMQ97735.1 | hypothetical protein [Solanum melongena] |
| EGP03975 | YP_006503776.1 | ATP synthase CF1 alpha subunit [Datura stramonium] |
| EGP03976 | AKZ21515.1 | ATP synthase CF1 alpha subunit, partial [Nepeta cataria] |
| EGP04188 | KAA0063014.1 | Photosystem II CP47 reaction center protein [Cucumis melo var. makuwa] |
| EGP04212 | YP_009751902.1 | photosystem II 43 kDa protein [Cyclanthera pedata] |
| EGP05980 | AKQ98361.1 | ferredoxin [Nicotiana benthamiana] |
| EGP06158 | XP_004230618.1 | psbP domain-containing protein 1, chloroplastic isoform X1 [Solanum lycopersicum] |
| EGP07155 | XP_016552228.1 | PREDICTED: ferredoxin, root R-B2-like [Capsicum annuum] |
| EGP07759 | XP_006340475.1 | PREDICTED: uncharacterized protein LOC102579691 [Solanum tuberosum] |
| EGP07767 | YP_009587247.1 | AtpA [Solanum kurtzianum] |
| EGP07851 | XP_004239534.1 | uncharacterized protein LOC101251983 [Solanum lycopersicum] |
| EGP07930 | AGJ51243.1 | ATP synthase CF0 B subunit [Solanum carolinense] |
| EGP08667 | TEY36774.1 | photosystem I P700 chlorophyll a apoprotein A2 [Salvia splendens] |
| EGP09306 | TMW81301.1 | hypothetical protein EJD97_010507 [Solanum chilense] |
| EGP09799 | XP_006343078.1 | PREDICTED: photosystem I reaction center subunit VI, chloroplastic-like [Solanum tuberosum] |
| EGP10305 | XP_006345209.2 | PREDICTED: psbQ-like protein 3, chloroplastic [Solanum tuberosum] |
| EGP10312 | XP_006345107.1 | PREDICTED: psbP-like protein 1, chloroplastic [Solanum tuberosum] |
| EGP10784 | YP_009254084.1 | AtpE [Solanum melongena] |
| EGP10785 | YP_009515855.1 | ATP synthase CF1 beta subunit [Solanum polhillii] |
| EGP11863 | OTF84393.1 | putative photosynthetic reaction centre, L/M, Photosystem antenna protein-like protein [Helianthus annuus] |
| EGP11967 | QEM01494.1 | hypothetical protein [Solanum tuberosum] |
| EGP12010 | YP_009336173.1 | photosystem II protein D1 [Ulmus macrocarpa] |
| EGP12011 | QBE88477.1 | photosystem II protein D1 [Lophiocarpus polystachyus] |
| EGP12426 | ALI90409.1 | PsaB, partial [Phytocrene racemosa] |
| EGP12941 | XP_015070726.1 | ferredoxin C 1, chloroplastic [Solanum pennellii] |
| EGP13061 | XP_006352721.1 | PREDICTED: photosystem I reaction center subunit IV A, chloroplastic [Solanum tuberosum] |
| EGP13122 | XP_006366818.1 | PREDICTED: photosystem I reaction center subunit XI, chloroplastic-like [Solanum tuberosum] |
| EGP13123 | XP_006366818.1 | PREDICTED: photosystem I reaction center subunit XI, chloroplastic-like [Solanum tuberosum] |
| EGP13424 | XP_004241330.1 | photosystem I subunit O [Solanum lycopersicum] |
| EGP13945 | XP_016578621.1 | PREDICTED: photosystem I reaction center subunit VI, chloroplastic [Capsicum annuum] |
| EGP14367 | ABF72923.2 | chloroplast chlorophyll a/b-binding protein [Solanum nigrum] |
| EGP14522 | AVA29993.1 | photosystem II protein D1 [Lansium domesticum] |
| EGP14523 | YP_009365640.1 | photosystem II protein D1 precursor [Digitalis lanata] |
| EGP14524 | QCD95922.1 | photosystem I chlorophyll a apoprotein A1 [Vigna unguiculata] |
| EGP14556 | XP_006356675.1 | PREDICTED: photosystem I reaction center subunit II, chloroplastic [Solanum tuberosum] |
| EGP16312 | XP_019267783.1 | PREDICTED: uncharacterized protein LOC109245053 [Nicotiana attenuata] |
| EGP16878 | QBB20364.1 | chloroplast photosystem II reaction center W protein, partial [Withania somnifera] |
| EGP17024 | XP_009762297.1 | PREDICTED: uncharacterized protein LOC104214342 [Nicotiana sylvestris] |
| EGP17031 | XP_009803380.1 | PREDICTED: photosystem II reaction center Psb28 protein [Nicotiana sylvestris] |
| EGP17425 | TYI09251.1 | hypothetical protein ES332_A09G058700v1 [Gossypium tomentosum] |
| EGP17662 | NP_054484.1 | ATP synthase CF0 A subunit [Nicotiana tabacum] |
| EGP17663 | AAA84683.1 | ATPase subunit I [Nicotiana tabacum] |
| EGP17664 | AGJ51242.1 | ATP synthase CF1 alpha subunit [Solanum carolinense] |
| EGP17909 | RHN59703.1 | putative photosystem II [Medicago truncatula] |
| EGP18988 | ESQ45945.1 | hypothetical protein EUTSA_v10011087mg [Eutrema salsugineum] |
| EGP19810 | OMP13956.1 | hypothetical protein COLO4_00557 [Corchorus olitorius] |
| EGP19864 | AAX57356.1 | putative ferredoxin-NADP reductase, partial [Solanum peruvianum] |
| EGP19969 | AGJ51243.1 | ATP synthase CF0 B subunit [Solanum carolinense] |
| EGP19970 | YP_009254063.1 | AtpA [Solanum melongena] |
| EGP20293 | PHT29346.1 | ATP synthase subunit alpha, chloroplastic [Capsicum baccatum] |
| EGP20423 | YP_004286003.1 | photosystem I P700 apoprotein A2 [Gossypium thurberi] |
| EGP20424 | YP_009523363.1 | cytochrome f [Solanum glabratum] |
| EGP20596 | YP_009774948.1 | photosystem II 44 kDa protein [Eleocharis dulcis] |
| EGP20597 | KEH17712.1 | photosystem I P700 chlorophyll A apoprotein A2 [Medicago truncatula] |
| EGP20598 | YP_009567990.1 | photosystem I P700 apoprotein A1 [Fallopia multiflora] |
| EGP20599 | AKZ22623.1 | photosystem I P700 chlorophyll a apoprotein A1 [Solanum rostratum] |
| EGP20749 | XP_016548442.1 | PREDICTED: photosystem I reaction center subunit psaK, chloroplastic [Capsicum annuum] |
| EGP20975 | XP_006352219.1 | PREDICTED: photosystem I reaction center subunit N, chloroplastic [Solanum tuberosum] |
| EGP21173 | XP_006364767.1 | PREDICTED: photosystem II core complex proteins psbY, chloroplastic [Solanum tuberosum] |
| EGP22013 | XP_006355121.1 | PREDICTED: photosynthetic NDH subunit of lumenal location 2, chloroplastic isoform X1 [Solanum tuberosum] |
| EGP22288 | XP_006367399.1 | PREDICTED: ferredoxin-like [Solanum tuberosum] |
| EGP22744 | AGJ51242.1 | ATP synthase CF1 alpha subunit [Solanum carolinense] |
| EGP23376 | XP_015060642.1 | photosystem II 10 kDa polypeptide, chloroplastic-like [Solanum pennellii] |
| EGP23441 | XP_006365954.1 | PREDICTED: ATP synthase gamma chain, chloroplastic-like [Solanum tuberosum] |
| EGP23675 | XP_015061236.1 | cytochrome b6-f complex iron-sulfur subunit, chloroplastic [Solanum pennellii] |
| EGP24079 | XP_006345545.1 | PREDICTED: ATP synthase subunit delta, chloroplastic [Solanum tuberosum] |
| EGP24533 | XP_015163558.1 | PREDICTED: ferredoxin, root R-B2-like [Solanum tuberosum] |
| EGP24700 | XP_016543391.1 | PREDICTED: oxygen-evolving enhancer protein 2, chloroplastic-like [Capsicum annuum] |
| EGP25075 | XP_028760969.1 | uncharacterized protein LOC114719625 [Prosopis alba] |
| EGP25088 | YP_009714350.1 | photosystem II 47 kDa protein [Paphiopedilum purpuratum] |
| EGP25236 | QFQ38839.1 | photosystem II CP47 chlorophyll apoprotein [Allomorphia balansae] |
| EGP25269 | YP_009254084.1 | AtpE [Solanum melongena] |
| EGP25271 | KAF5817508.1 | putative photosystem I PsaA/PsaB [Helianthus annuus] |
| EGP25272 | QFQ47127.1 | photosystem I P700 apoprotein A1 [Allomorphia sp. Liu 681] |
| EGP25275 | PHT25528.1 | ATP synthase subunit alpha, chloroplastic [Capsicum baccatum] |
| EGP25296 | OTF84537.1 | putative photosynthetic reaction centre, L/M, Photosystem antenna protein-like protein [Helianthus annuus] |
| EGP25297 | TKS04886.1 | Photosystem I P700 chlorophyll a apoprotein A1 [Populus alba] |
| EGP25438 | XP_009625635.1 | photosystem I reaction center subunit VI-2, chloroplastic-like [Nicotiana tomentosiformis] |
| EGP25632 | QEM01494.1 | hypothetical protein [Solanum tuberosum] |
| EGP25868 | XP_019245375.1 | PREDICTED: uncharacterized oxidoreductase At4g09670-like [Nicotiana attenuata] |
| EGP25871 | TMW88180.1 | hypothetical protein EJD97_018950 [Solanum chilense] |
| EGP26648 | XP_006352484.1 | PREDICTED: photosynthetic NDH subunit of lumenal location 3, chloroplastic [Solanum tuberosum] |
| EGP26932 | CAB89998.1 | ATP synthase beta subunit, partial [Solanum nodiflorum] |
| EGP26933 | YP_009254084.1 | AtpE [Solanum melongena] |
| EGP27429 | XP_009796636.1 | PREDICTED: ferredoxin [Nicotiana sylvestris] |
| EGP27648 | XP_006351160.1 | PREDICTED: uncharacterized protein LOC102590783 [Solanum tuberosum] |
| EGP28239 | ADM92638.1 | AtpA, partial [Franklinia alatamaha] |
| EGP28240 | PHT85293.1 | ATP synthase subunit alpha, chloroplastic [Capsicum annuum] |
| EGP28241 | YP_009913652.1 | ATP synthase CF0 C subunit [Luffa acutangula] |
| EGP28773 | XP_019244898.1 | PREDICTED: plastocyanin A'/A'' [Nicotiana attenuata] |
| EGP29046 | PHU17848.1 | Cytochrome f [Capsicum chinense] |
| EGP29117 | PHT78033.1 | Photosystem I chlorophyll a apoprotein A1 [Capsicum annuum] |
| EGP29118 | QHN96859.1 | Photosystem I P700 chlorophyll a apoprotein [Arachis hypogaea] |
| EGP29333 | AGJ51242.1 | ATP synthase CF1 alpha subunit [Solanum carolinense] |
| EGP29334 | YP_009254063.1 | AtpA [Solanum melongena] |
| EGP29506 | XP_006361933.1 | PREDICTED: ferredoxin--NADP reductase, leaf-type isozyme, chloroplastic-like [Solanum tuberosum] |
| EGP29507 | XP_006361933.1 | PREDICTED: ferredoxin--NADP reductase, leaf-type isozyme, chloroplastic-like [Solanum tuberosum] |
| EGP29746 | XP_016558935.1 | PREDICTED: oxygen-evolving enhancer protein 1, chloroplastic [Capsicum annuum] |
| EGP29789 | XP_015170483.1 | PREDICTED: uncharacterized protein LOC107063301 [Solanum tuberosum] |
| EGP30231 | XP_006353052.1 | PREDICTED: photosystem I reaction center subunit III, chloroplastic-like [Solanum tuberosum] |
| EGP30232 | XP_006353053.1 | PREDICTED: photosystem I reaction center subunit III, chloroplastic-like [Solanum tuberosum] |
| EGP30290 | XP_006362590.1 | PREDICTED: cytochrome c6, chloroplastic [Solanum tuberosum] |
| EGP30596 | TMW84604.1 | hypothetical protein EJD97_024849 [Solanum chilense] |
| EGP30637 | XP_006348117.1 | PREDICTED: ATP synthase gamma chain, chloroplastic [Solanum tuberosum] |
| EGP30917 | TMW93405.1 | hypothetical protein EJD97_011773 [Solanum chilense] |
| EGP31442 | TMW86777.1 | hypothetical protein EJD97_020899 [Solanum chilense] |
| EGP32120 | XP_004251055.1 | ferredoxin, root R-B1 [Solanum lycopersicum] |
| EGP32603 | XP_017644167.1 | PREDICTED: photosystem II CP47 reaction center protein [Gossypium arboreum] |
| EGP32661 | KAF4359983.1 | hypothetical protein F8388_004490 [Cannabis sativa] |
| EGP32873 | KCW64414.1 | hypothetical protein EUGRSUZ_G02034 [Eucalyptus grandis] |
| EGP33109 | YP_009254085.1 | AtpB [Solanum melongena] |
| EGP33113 | QCD95922.1 | photosystem I chlorophyll a apoprotein A1 [Vigna unguiculata] |
| EGP33199 | PHT78022.1 | Cytochrome f [Capsicum annuum] |
| EGP33262 | XP_015164254.1 | PREDICTED: uncharacterized protein LOC107060652 [Solanum tuberosum] |
| EGP33420 | XP_006360615.1 | PREDICTED: photosynthetic NDH subunit of lumenal location 1, chloroplastic [Solanum tuberosum] |
| EGP33962 | TMW81301.1 | hypothetical protein EJD97_010507 [Solanum chilense] |
| EGP34376 | YP_009254084.1 | AtpE [Solanum melongena] |
| EGP34377 | CAB90085.1 | ATP synthase beta subunit, partial [Gonocaryum litorale] |
| EGP34378 | YP_009515855.1 | ATP synthase CF1 beta subunit [Solanum polhillii] |
| EGP34382 | YP_009171879.1 | Ycf4 [Solanum nigrum] |
| EGP34383 | YP_009171880.1 | CemA [Solanum nigrum] |
| EGP34384 | YP_009171881.1 | PetA [Solanum nigrum] |
| EGP34385 | KAF3449782.1 | hypothetical protein FNV43_RR05860 [Rhamnella rubrinervis] |
| EGP34386 | NP_054526.1 | photosystem II 47 kDa protein [Nicotiana tabacum] |
| EGP34426 | YP_009123347.1 | photosystem II CP43 chlorophyll apoprotein [Iochroma nitidum] |
| EGP34428 | YP_009254077.1 | PsaB [Solanum melongena] |
| EGP34429 | YP_009254078.1 | PsaA [Solanum melongena] |
| EGP34642 | YP_009669716.1 | photosystem II subunit D1 [Ruizterania albiflora] |
| EGP34731 | QBE88477.1 | photosystem II protein D1 [Lophiocarpus polystachyus] |
| EGP34761 | YP_009254078.1 | PsaA [Solanum melongena] |
| EGP34762 | YP_009254077.1 | PsaB [Solanum melongena] |
| EGP34764 | YP_009123347.1 | photosystem II CP43 chlorophyll apoprotein [Iochroma nitidum] |
| EGP34806 | YP_009271276.1 | photosystem II protein D1 [Amaranthus hypochondriacus] |
| EGP34814 | YP_009108423.1 | photosystem II protein D1 [Genlisea margaretae] |
| EGP34815 | YP_009155294.1 | photosystem I P700 chlorophyll a apoprotein A1 [Seseli montanum] |
| EGP34820 | YP_009254085.1 | AtpB [Solanum melongena] |
| EGP34829 | YP_009651660.1 | photosystem I P700 apoprotein A1 [Dillenia indica] |
| EGP34831 | YP_009651660.1 | photosystem I P700 apoprotein A1 [Dillenia indica] |
| Solanum_melongena_newGene_1443 | YP_009419690.1 | photosystem II p680 chlorophyll A apoprotein CP-47 [Anneslea fragrans] |
| Solanum_melongena_newGene_1502 | XP_021732719.1 | uncharacterized protein LOC110699535 [Chenopodium quinoa] |
| Solanum_melongena_newGene_3079 | YP_009252621.1 | cytochrome b6 [Iochroma lehmannii] |
| Solanum_melongena_newGene_4039 | XP_028767563.1 | uncharacterized protein LOC114725246 [Prosopis alba] |
| Solanum_melongena_newGene_5436 | XP_028760969.1 | uncharacterized protein LOC114719625 [Prosopis alba] |
| Solanum_melongena_newGene_6534 | XP_028760969.1 | uncharacterized protein LOC114719625 [Prosopis alba] |
| Solanum_melongena_newGene_7098 | XP_028760969.1 | uncharacterized protein LOC114719625 [Prosopis alba] |
| Solanum_melongena_newGene_7162 | OMO99037.1 | hypothetical protein CCACVL1_03959 [Corchorus capsularis] |
| Solanum_melongena_newGene_8173 | PHT45141.1 | Photosystem I chlorophyll a apoprotein A2 [Capsicum baccatum] |
| Solanum_melongena_newGene_985 | EYU39701.1 | hypothetical protein MIMGU_mgv11b022363mg, partial [Erythranthe guttata] |

Table S4 Primer sequences for qRT-PCR experiments.

| **Primer name** | **Primer sequence (5'to3')** |
| --- | --- |
| EGP18530-SnRK2-F | AGATGGTGTCGCAAGGTG |
| EGP18530-SnRK2-R | CGAGCGTGAGGGAGTAAAT |
| EGP11066-PP2C-F | TCAGGGTAGTAGGGATAA |
| EGP11066-PP2C-R | AACCAAGGCAAGTAACAG |
| EGP18001-AUX1-F | TCACCCTAACACTACCCT |
| EGP18001-AUX1-R | TCCCTATAACTTTCTCCC |
| EGP14445-AUX1/IAA-F | AGGTTTACCAGGGAGGAC |
| EGP14445-AUX1/IAA-R | CACGCTAACTTTGACATACA |
| EGP01302-ARF19-F | GCGATTTCGGATGATGTTTGA |
| EGP01302-ARF19-R | CTGTGAGCCTTTCCATCGTA |
| EGP14379- CYP90B -F | CTCTCAATGCGTGGTCCTTC |
| EGP14379- CYP90B -R | ATGTCGAACCCCCTTTATCAC |
| EGP20989-MYB-F | TAGGTGGTCACTCATAGC |
| EGP20989-MYB-R | GTTTGTCCGTAGGTTTCT |
| EGP06499-SQS-F | GCTTCCTCGGAGATTGTGGA |
| EGP06499-SQS-R | CGAGTGAGATCTCCGAAAGA |
| EGP09657-C2H2-F | ATCAGCTTAAGCCCGGATCA |
| EGP09657-C2H2-R | ATGGAGCTCTAACAAGTGCC |
| EGP25367-LRK10L-2.6-F | TTGGAGGAGTGTCACACAAA |
| EGP25367-LRK10L-2.6-R | TCTTTCGGCGCTGTAGTTTT |
| EGP00263-DELLA (RGL1)-F | ACATTAAGTGATAGCCTGGAGC |
| EGP00263-DELLA (RGL1)-R | AAGCAGTAGACTGTTATGGCG |
| EGP09992-CYP90B-F | ATCATGGCAAGCGATGCAAG |
| EGP09992-CYP90B-R | AGCCAAAGAGATTTCCAACCT |
| EGP06774-GST-F | ACGAGATGTCGGAAGTGA |
| EGP06774-GST-R | TTAGGCAAGATAGGAGGG |
| EGP15357-TreY-F | CCAATGGGAGGAGTCTGT |
| EGP15357-TreY-R | CCAATGGGAGGAGTCTGT |
| EGP28780-AMY-F | AACGACGGAAGCGACGAA |
| EGP28780-AMY-R | AGCCCACTCCAAGAAATCAACT |
| EGP23490-AMY-F | TGGCGAGATTTGATACAG |
| EGP23490-AMY-R | TGAACGAAAGCTCCCTTA |


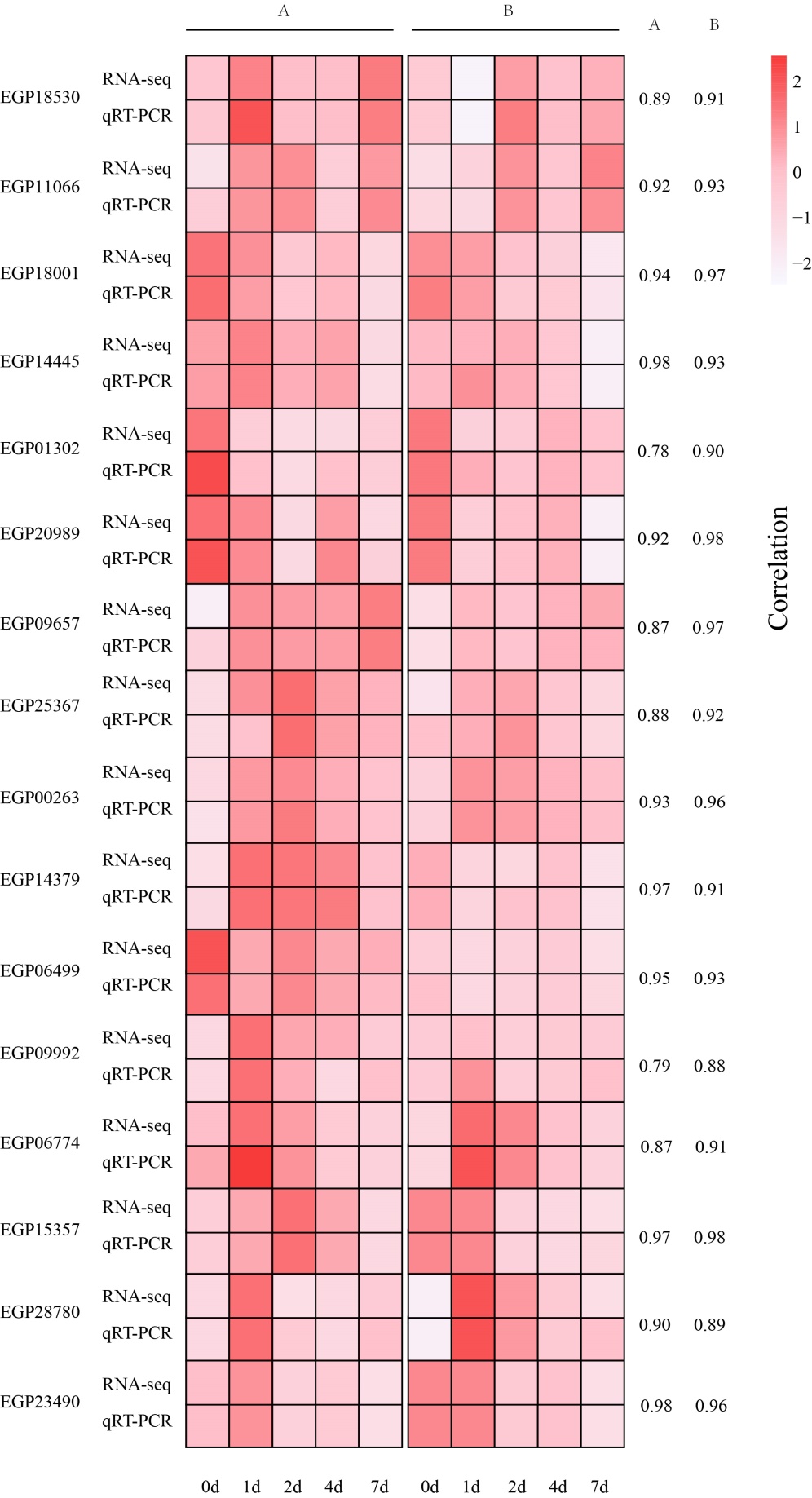


Figure S4 Correlation analysis of the results of qRT PCR validation of RNA Seq data.
